# ZBP1-driven pyroptosis-associated alveolar macrophages exacerbate epithelial dysfunction in sepsis

**DOI:** 10.1038/s41419-026-09043-y

**Published:** 2026-06-26

**Authors:** Feicheng Zhou, Haolin Tian, Haixia Wang, Hongxiang Huang, Xuwei Lin, Yuanyuan Zhao, Huaijun Chen, Ting Gong

**Affiliations:** 1 Department of Anesthesiology, Shenzhen Hospital, Southern Medical University, Shenzhen, China; 2 Shenzhen School of Clinical Medicine, Southern Medical University, Shenzhen, China; 3 Department of Critical Care Medicine, Peking University Shenzhen Hospital, Shenzhen, China; 4 Department of Neurosurgery, The Second Affiliated Hospital, Zhejiang University School of Medicine, Hangzhou, China; 5 Key Laboratory of Precise Treatment and Clinical Translational Research of Neurological Diseases, Hangzhou, China

**Keywords:** Apoptosis, Organelles

## Abstract

The lung is highly vulnerable to inflammatory injury during sepsis, and acute lung injury (ALI) is a major cause of mortality in critically ill patients. Pyroptosis amplifies immune responses by promoting the release of inflammatory cytokines, and Z-DNA binding protein 1 (ZBP1) has emerged as a key upstream regulator of programmed cell death and inflammatory signaling. Nevertheless, the contribution of ZBP1 to human sepsis-induced ALI and its associated cellular programs remains poorly defined. Here, by integrating single-cell RNA sequencing data from bronchoalveolar lavage fluid (BALF) of patients with sepsis-induced ALI and from septic mouse lungs, we identified a distinct subset of pyroptosis-associated macrophages that expands during disease progression and exhibits ZBP1-dependent inflammasome activation. ZBP1 activation promoted inflammasome assembly, induced macrophage pyroptosis, and released pro-inflammatory mediators that impaired mitochondrial function and barrier integrity of alveolar type II (AT2) epithelial cells. ZBP1 deficiency markedly attenuated macrophage-AT2 inflammatory signaling and reduced the inflammatory amplification loop. Collectively, these findings identify ZBP1-mediated macrophage pyroptosis as a critical mechanism driving epithelial dysfunction during sepsis-induced ALI and provide a rationale for developing ZBP1-targeted strategies to restore immune–epithelial homeostasis and prevent organ failure in sepsis.

**Figure:**
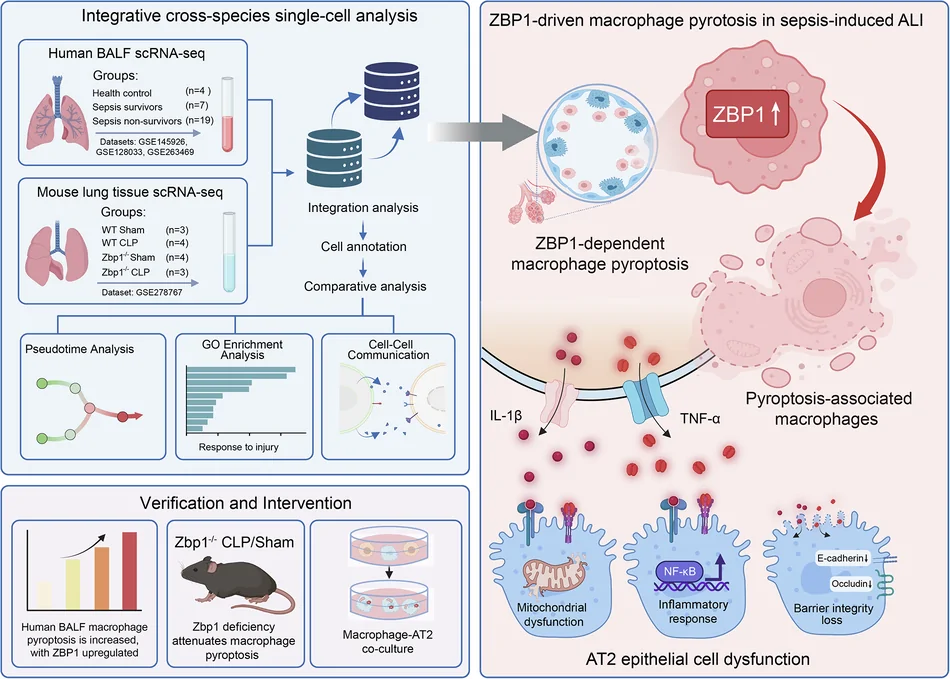
In sepsis-induced acute lung injury, ZBP1 drives macrophage pyroptosis and amplifies inflammatory signaling, thereby promoting mitochondrial dysfunction, inflammatory activation, and barrier integrity loss in AT2 epithelial cells. Zbp1 deficiency suppresses macrophage pyroptosis, weakens macrophage-epithelial inflammatory crosstalk, and mitigates epithelial injury.

In sepsis-induced acute lung injury, ZBP1 drives macrophage pyroptosis and amplifies inflammatory signaling, thereby promoting mitochondrial dysfunction, inflammatory activation, and barrier integrity loss in AT2 epithelial cells. Zbp1 deficiency suppresses macrophage pyroptosis, weakens macrophage-epithelial inflammatory crosstalk, and mitigates epithelial injury.

## Introduction

Sepsis is a life-threatening organ dysfunction caused by a dysregulated host response to infection and remains the leading cause of death among critically ill patients in intensive care units [1]. Globally, an estimated 48.9 million annual sepsis cases and 11.0 million related deaths account for 19.7% of all global deaths [2]. The lung is particularly vulnerable to the excessive inflammatory response during sepsis, often developing acute lung injury (ALI) at an early stage. The mortality of sepsis-induced ALI approaches 40%, making it a major contributor to sepsis-related deaths and a serious threat to human health [3]. Pathologically, ALI is characterized by diffuse interstitial and alveolar edema caused by injury to pulmonary endothelial and alveolar epithelial cells, leading to hypoxemic respiratory failure or permeability pulmonary edema that may progress to acute respiratory distress syndrome. Its development involves excessive immune activation and the release of inflammatory mediators, particularly from infiltrating macrophages that aggravate lung injury [4, 5].

Pyroptosis has gained increasing attention for its pivotal role in the pathogenesis of various infectious and inflammatory diseases [6, 7]. Inflammasomes serve as cytosolic surveillance complexes that detect microbial components and activate inflammatory caspases, primarily caspase-1 and caspase-11 [8]. This activation drives the cleavage of gasdermin D (GSDMD) and the subsequent formation of plasma membrane pores, resulting in osmotic lysis and the release of interleukin-1β (IL-1β) and interleukin-18 (IL-18) [7, 9]. Although inflammasome activation and pyroptosis are essential for host defense against pathogens, excessive activation can lead to uncontrolled inflammation, multi-organ dysfunction, and even death [10, 11]. While macrophage pyroptosis has been implicated in sepsis-associated tissue injury, its role in mediating epithelial dysfunction and the molecular mechanisms connecting these events remain incompletely understood.

Z-DNA binding protein 1 (ZBP1) is an innate immune sensor that recognizes double-stranded nucleic acids adopting the Z conformation, including Z-DNA and Z-RNA [12, 13]. The Zα domain of ZBP1 undergoes conformational rearrangement after binding to nucleic acid ligands, which triggers downstream signal transduction [14, 15]. ZBP1 interacts with RIPK1 and RIPK3 through its receptor-interacting protein homotypic interaction motif (RHIM), thereby regulating programmed cell death signaling pathways [16, 17]. In macrophages, interferon-inducible ZBP1 has been implicated in inflammatory signaling and immune activation [18]. Previous studies have further shown that ZBP1 links innate immune sensing to inflammatory cell death and cytokine-mediated immune activation during infection and tissue injury [5, 16, 19]. These findings suggest that ZBP1 may be particularly relevant to macrophage-driven inflammatory amplification in sepsis-induced ALI [20]. However, whether ZBP1 defines a pathogenic pyroptosis-associated alveolar macrophage program and contributes to macrophage–AT2 epithelial cell crosstalk in sepsis-induced ALI remains insufficiently understood. Single-cell RNA sequencing provides a useful approach to characterize these ZBP1-associated cellular alterations and intercellular communication networks at single-cell resolution [20, 21].

In this study, we applied single-cell transcriptomic analysis to comprehensively characterize cellular alterations associated with sepsis-induced ALI. By integrating human bronchoalveolar lavage fluid (BALF) datasets and mouse lung single-cell transcriptomes, we aimed to delineate the cellular heterogeneity and immune regulatory mechanisms underlying macrophage activation and pyroptosis. We further performed ligand–receptor interaction analysis to map the macrophage-centered intercellular communication network contributing to epithelial dysfunction. Finally, our findings from the single-cell analysis were validated through in vitro experiments and studies using *Zbp1* knockout mice. Collectively, this study reveals the pivotal role of macrophages, particularly ZBP1-mediated pyroptosis, in regulating macrophage–epithelial crosstalk during sepsis-induced ALI and provides new insights into potential therapeutic strategies for sepsis-related lung injury.

## Results

### Escalating macrophage pyroptosis signatures across sepsis progression

**Fig. 1:**
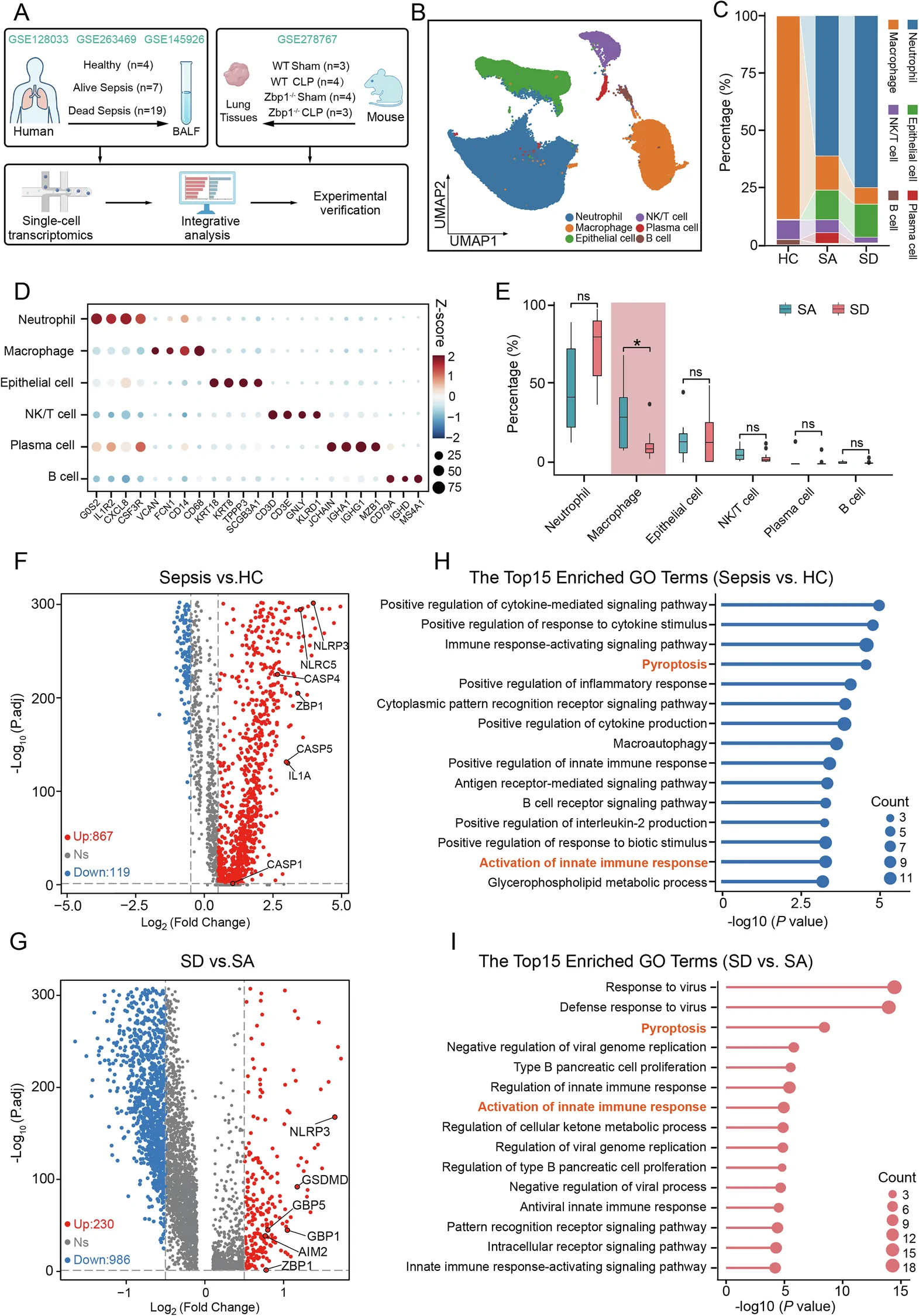
Integrative single-cell transcriptomic analysis identifies macrophage alterations and pyroptosis-related signatures across healthy controls, sepsis survivors, and non-survivors. **A** Schematic workflow of the study. Single-cell RNA sequencing data from human bronchoalveolar lavage fluid (BALF) and mouse lung single-cell datasets were integrated to explore cellular dynamics and transcriptional programs associated with sepsis-induced acute lung injury, followed by experimental validation. **B** UMAP visualization of major cell populations, including macrophages, epithelial cells, neutrophils, natural killer/T cells, plasma cells, and B cells. **C** Stacked bar plots showing the distribution of each cell type across HC, SA, and SD. **D** Dot plot displaying canonical marker genes used to confirm the identity of each cluster. **E** Quantitative comparison of immune and epithelial cells identified from BALF between SA and SD groups. **F** Volcano plot showing differentially expressed genes (DEGs) between sepsis (SA and SD) and HC. **G** Volcano plot showing DEGs between SD and SA. **H** Gene Ontology (GO) enrichment analysis of DEGs between sepsis and HC, indicating activation of inflammatory response, cytokine signaling, and cell death pathways. **I** GO enrichment analysis of DEGs between SD and SA. Data are presented as mean ± SEM. ^*^P < 0.05, ^**^P < 0.01, ^***^P < 0.001.

To investigate cellular alterations associated with sepsis-induced ALI, we performed an integrative single-cell transcriptomic analysis by combining human BALF datasets, mouse lung single-cell transcriptomes from public transcriptomic data (GSE128033, GSE263469, GSE145926, and GSE278767). Human BALF samples were categorized into three groups: healthy controls (HC, *n* = 4), sepsis survivors (SA, *n* = 7), and sepsis non-survivors (SD, *n* = 19). Correspondingly, mouse lung tissues from wild-type (WT) and *Zbp1*^−/−^ mice under Sham or CLP conditions were integrated for cross-species comparative analysis. This workflow enabled the identification of shared immune cell alterations and conserved pyroptosis-related signatures between human and mouse sepsis models (Fig. 1A).

After quality control and batch correction, unsupervised clustering and UMAP visualization identified six major cell populations, including macrophages, epithelial cells, neutrophils, natural killer T (NKT) cells, plasma cells, and B cells (Fig. 1B). Canonical marker genes were used to confirm cluster identity (Fig. 1D).

Comparison of cellular composition across clinical groups revealed dynamic cellular shifts during disease progression (Fig. 1C). Notably, neutrophils and epithelial cells increased from HC to SA and were most abundant in the SD group, whereas macrophages declined in SD. To further validate these findings in the clinical setting, we quantified BALF-derived cell populations in SA and SD patients. Among immune and epithelial compartments, macrophages exhibited the most pronounced difference between the two groups, suggesting their potential involvement in the progression to fatal outcomes (Fig. 1E).

To explore underlying molecular changes, we next performed differential gene expression analysis. Compared with HC, sepsis patients displayed significant upregulation of inflammatory and pyroptosis-related genes, including ZBP1, NLRP3, and CASP1 (Fig. 1F). When comparing SD with SA, these cell death and immune activation genes were further elevated in non-survivors (Fig. 1G). Gene Ontology enrichment analysis demonstrated that sepsis patients were enriched in inflammatory response, cytokine signaling, and programmed cell death pathways compared to HC (Fig. 1H). Importantly, SD showed enhanced enrichment of pyroptosis, viral response, and ZBP1-associated signaling pathways compared to SA (Fig. 1I), indicating that pyroptosis-related programs are strongly activated in patients with fatal outcomes.

Collectively, these results identify macrophages as a major cell population altered during sepsis progression and reveal pyroptosis-related transcriptional activation, including ZBP1 upregulation, as a key molecular signature in non-survivors, thereby implicating macrophage pyroptosis in the development of severe lung injury.

### Pyroptosis-associated NLRP3^+^ macrophages enriched in non-survivors and exhibit ZBP1-dependent inflammasome activation

**Fig. 2:**
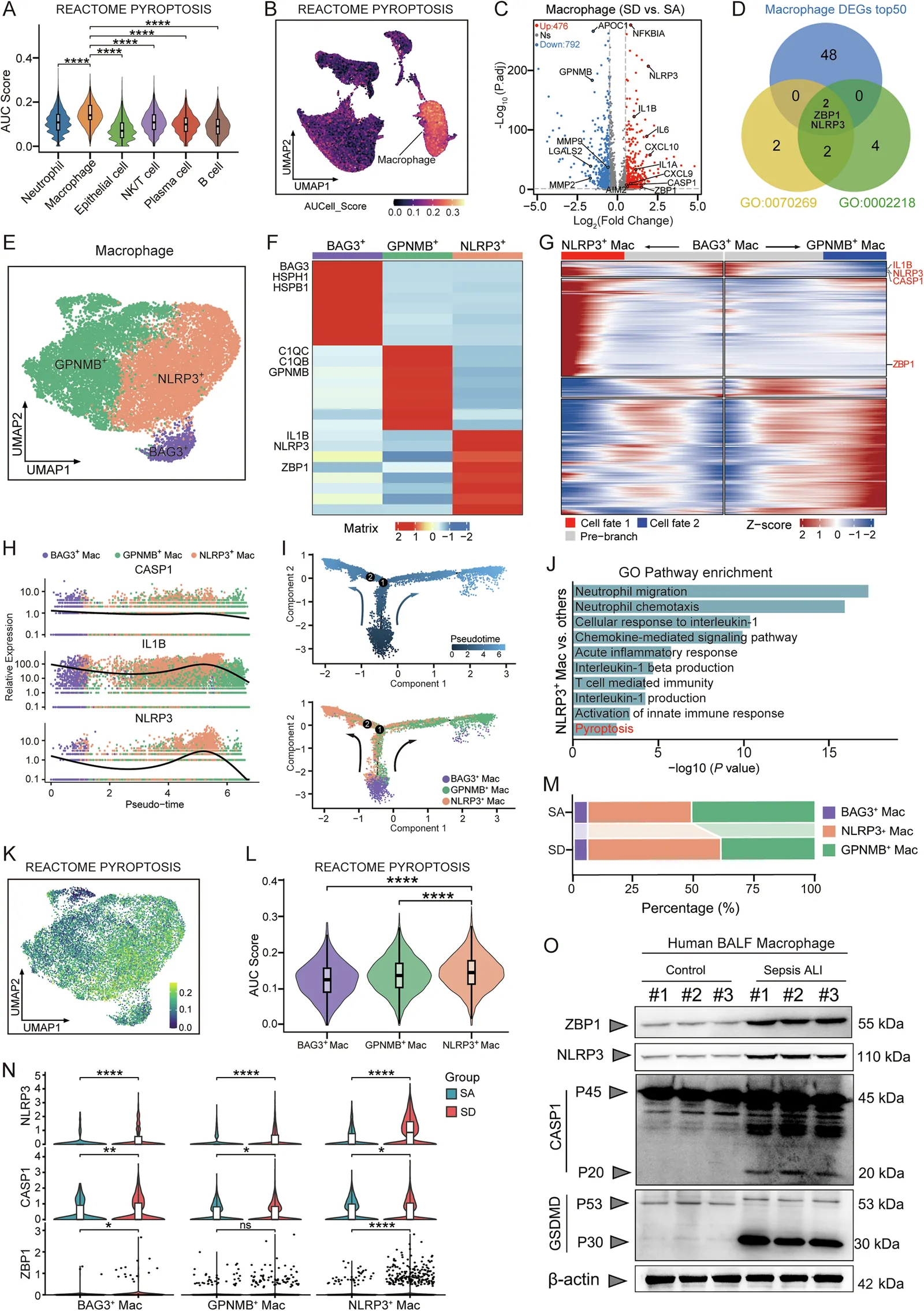
NLRP3^+^ macrophages represent a pyroptosis-associated subset enriched in non-survivors. **A** Violin plots showing the pyroptosis signaling score across major cell types, with macrophages displaying the highest pyroptosis levels. **B** UMAP visualization highlighting that high pyroptosis activity is predominantly localized within macrophages. **C** Volcano plot of differentially expressed genes (DEGs) in macrophages between SD and SA groups, revealing upregulation of pyroptosis- and inflammasome-related genes, including ZBP1, NLRP3, IL1B, and CASP1. **D** Venn diagram of the top 50 macrophage DEGs enriched in key GO terms, identifying ZBP1 and NLRP3 as shared hub genes. **E** UMAP plot showing three macrophage subsets (BAG3⁺, GPNMB⁺, and NLRP3⁺). **F** Heatmap of representative genes in each subset, demonstrating that the NLRP3⁺ macrophage subset expresses high levels of inflammasome and pyroptosis-related genes. **G** Pseudotime analysis illustrating dynamic expression of IL1B, NLRP3, CASP1, and ZBP1 along the macrophage trajectory. **H** Expression trends of CASP1, IL1B, and NLRP3 along pseudotime. **I** Trajectory plots showing that the NLRP3⁺ macrophage subset occupies the terminal branch associated with a pyroptotic fate. **J** GO enrichment analysis comparing NLRP3⁺ macrophages with other subsets, revealing enrichment of innate immune activation, IL-1 signaling, and pyroptosis pathways. **K**, **L** Pyroptosis signaling scores in macrophages and across macrophage subsets, showing that NLRP3⁺ macrophages have the highest pyroptosis activity. **M** Proportion of macrophage subsets in SA and SD BALF samples, with NLRP3⁺ macrophages markedly expanded in SD. **N** Expression levels of pyroptosis-related genes (CASP1, IL1B, NLRP3, ZBP1) in each macrophage subset, showing further upregulation in SD. **O** Western blot analysis of BALF-derived macrophages from healthy controls and patients with sepsis-induced ALI, showing increased expression of ZBP1, NLRP3, cleaved CASP1 (p20), and cleaved GSDMD (p30/p53) in sepsis-induced ALI. Data are presented as mean ± SEM. ^*^P < 0.05, ^**^P < 0.01, ^***^P < 0.001, ^****^P < 0.0001.

Given the prominent macrophage alterations observed in Fig. 1, we next investigated whether macrophages were the main contributors to pyroptosis during sepsis. Pyroptosis pathway scoring using AUCell revealed that macrophages had the highest pyroptosis activity among all major cell types (Fig. 2A), and UMAP mapping confirmed that pyroptosis signals were predominantly localized within macrophages (Fig. 2B). Differential expression analysis of macrophages between SD and SA showed significant upregulation of inflammasome- and pyroptosis-related genes, including ZBP1, NLRP3, IL1B, and CASP1 (Fig. 2C). A Venn diagram of top macrophage differentially expressed genes (DEGs) linked to key GO pathways further identified ZBP1 and NLRP3 as shared hub genes (Fig. 2D).

To determine whether specific macrophage populations drive pyroptosis, we re-clustered macrophages into three subsets: BAG3⁺, GPNMB⁺, and NLRP3⁺ (Fig. 2E). NLRP3⁺ macrophage subset exhibited the highest expression of inflammasome and pyroptosis-related genes (Fig. 2F). Pseudotime trajectory analysis further revealed a progressive increase in IL1B, NLRP3, CASP1, and ZBP1 along the differentiation path (Fig. 2G, H), and the NLRP3⁺ subset occupied the terminal branch associated with a pyroptotic fate (Fig. 2I). GO enrichment analysis confirmed that NLRP3^+^ macrophages were strongly enriched in innate immune activation, IL-1 signaling, and pyroptosis pathways (Fig. 2J), indicating that this subset represents the pyroptosis-associated macrophage population.

Pyroptosis scoring across macrophage subsets demonstrated that NLRP3⁺ macrophages had the highest pyroptosis activity (Fig. 2K, L). Importantly, the proportion of NLRP3⁺ macrophages was markedly increased in SD compared with SA (Fig. 2M), suggesting that expansion of this pyroptotic subset is associated with fatal outcomes. Consistent with this, the pyroptosis-related gene, moreover, KEGG analysis demonstrated genes, including CASP1, NLRP3, and ZBP1, were significantly upregulated in NLRP3⁺ macrophages from SD patients (Fig. 2N).

To validate these findings, we isolated macrophages from BALF of healthy controls and sepsis-induced ALI patients and examined key pyroptosis proteins. Western blot analysis confirmed increased expression of ZBP1 and NLRP3, cleavage of CASP1 (p20), and cleavage of GSDMD (p30/p53) in sepsis patients (Fig. 2O), directly demonstrating activation of macrophage pyroptosis in clinical samples. Together, these results identify the NLRP3⁺ macrophage subset as the pyroptosis-associated macrophage population that is expanded in non-survivors and displays ZBP1-dependent inflammasome activation.

### AT2 epithelial cells accumulate in BALF of non-survivors and display the strongest inflammatory activation

**Fig. 3:**
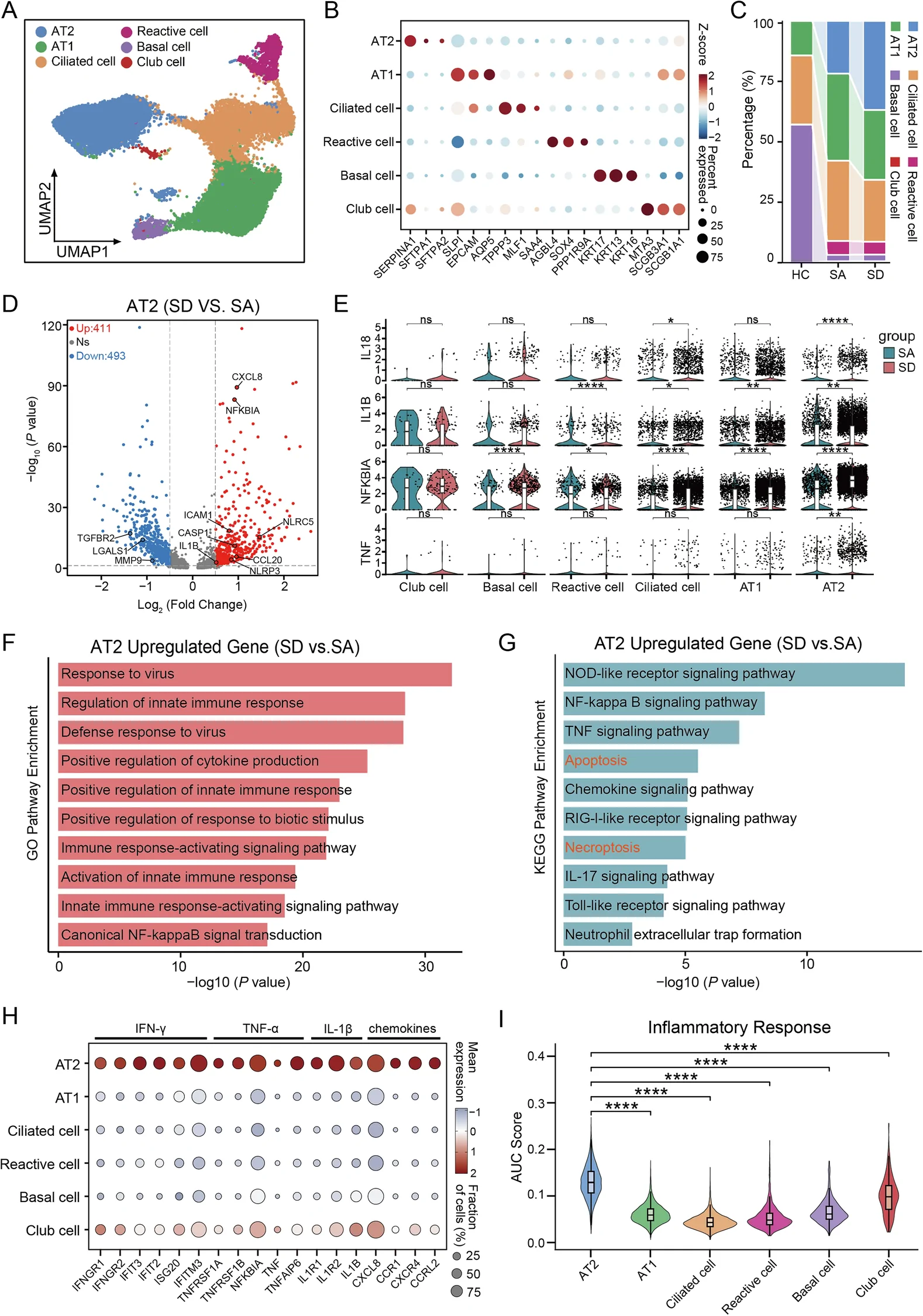
AT2 cells accumulate in BALF of non-survivors and exhibit the strongest inflammatory and cell death–related transcriptional responses among epithelial subtypes. **A** UMAP visualization of epithelial cell populations, including alveolar type 2 (AT2), type 1 (AT1), ciliated, basal, club, and reactive cells. **B** Dot plot of canonical marker genes confirming the identity of each epithelial subset. **C** Proportional distribution of epithelial subsets across HC, SA, and SD. AT2 cells were markedly increased in the SD group, indicating enhanced AT2 detachment and accumulation in BALF during severe injury. **D** Volcano plot of DEGs in AT2 cells between SD and SA, revealing upregulation of inflammatory and injury-related genes (IL1B, CXCL8, NFKBIA, CASP1, NLRP3). **E** Expression of inflammatory mediators (IL-18, IL-1β, NFKBIA, TNF) across epithelial subsets, showing that AT2 cells have the highest induction in SD. **F** GO enrichment of upregulated genes in AT2 cells (SD vs SA). **G** KEGG pathway enrichment of AT2 upregulated genes. **H** Dot plot of inflammatory cytokines and chemokines across epithelial subsets. **I** AUCell-based inflammatory response scores across epithelial subsets. Data are presented as mean ± SEM. ^*^P < 0.05, ^**^P < 0.01, ^***^P < 0.001, ^****^P < 0.0001.

To determine how epithelial cells respond during sepsis-induced lung injury, we reclustered epithelial populations into AT2, AT1, ciliated, basal, club, and reactive cells (Fig. 3A), and confirmed their identities with canonical markers (Fig. 3B). Comparison of cell proportions revealed marked redistribution of the epithelial landscape across disease progression. Notably, AT2 cells were significantly increased in the SD group compared with HC and SA (Fig. 3C), indicating enhanced AT2 detachment and accumulation in BALF during severe lung injury. This suggested that AT2 cells are highly vulnerable to injury in non-survivors.

Differential expression analysis of AT2 cells between SD and SA revealed strong induction of inflammatory and injury-related genes, including IL1B, CXCL8, NFKBIA, CASP1, and NLRP3 (Fig. 3D). Consistently, inflammatory mediators such as IL-18, IL-1β, NFKBIA, and TNF were most highly expressed in AT2 cells, especially in SD patients (Fig. 3E). GO enrichment confirmed activation of innate immune response, cytokine production, and NF-κB signaling pathways (Fig. 3F). Moreover, KEGG analysis demonstrated enrichment of apoptosis and necroptosis, as well as NOD-like receptor and TNF signaling pathways (Fig. 3G).

Furthermore, AT2 cells exhibited the highest expression of IFN-γ, TNF-α, IL-1β, and chemokines compared with other epithelial subsets (Fig. 3H), and scored the highest for inflammatory response activity (Fig. 3I).

Collectively, these findings indicate that AT2 cells are the most injury-prone epithelial population in non-survivors, characterized by increased BALF accumulation and simultaneous activation of inflammatory, apoptosis, and necroptosis pathways.

### Pyroptotic alveolar macrophage-derived TNF and IL-1 signaling mediates mitochondrial dysfunction and epithelial injury during sepsis-induced ALI

**Fig. 4:**
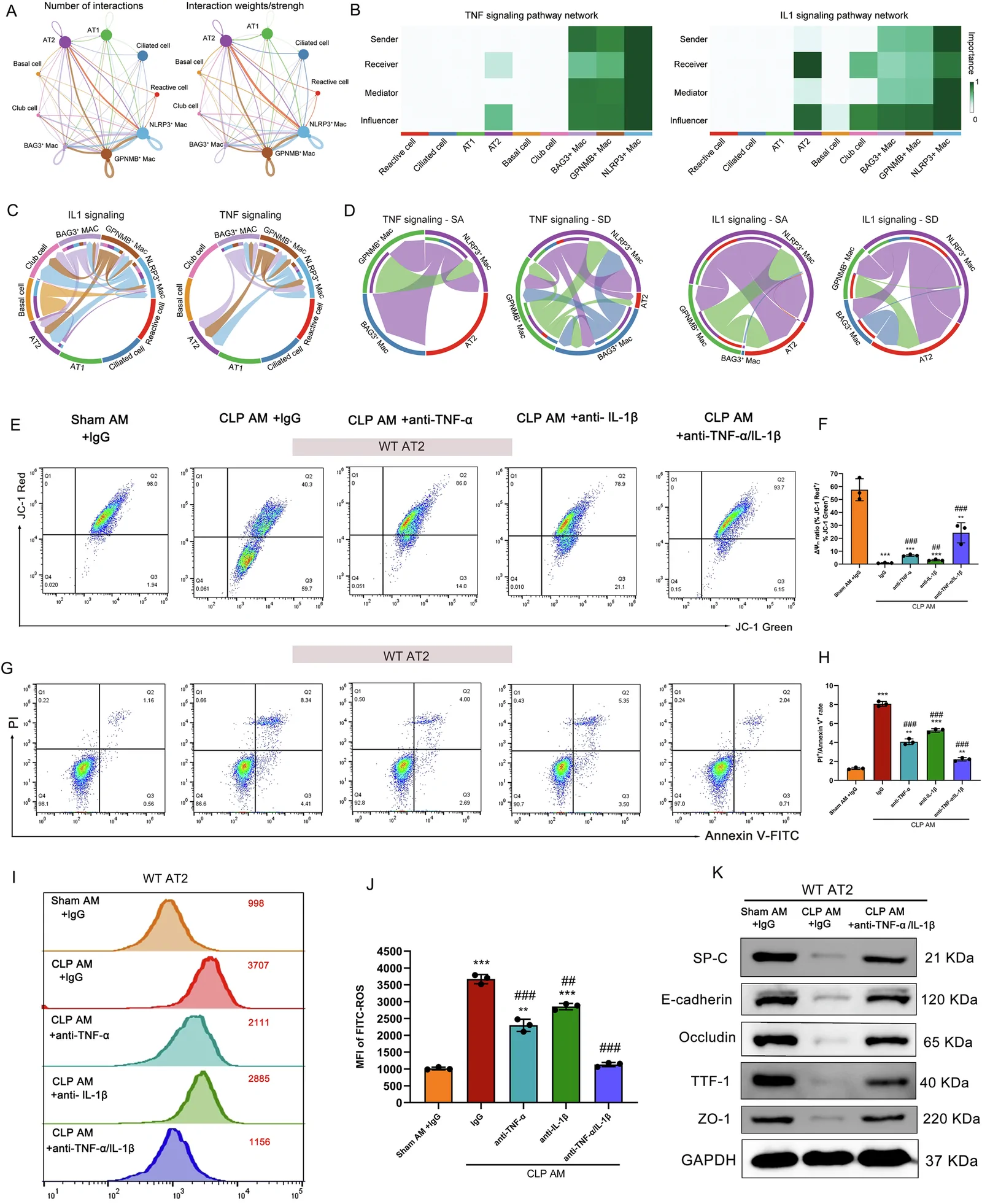
Pyroptotic alveolar macrophage-derived TNF and IL-1 signaling mediates mitochondrial dysfunction and epithelial injury in sepsis. **A** Cell-cell communication networks showing the number of interactions and interaction weights/strength among macrophage subsets, including BAG3⁺, GPNMB⁺, and NLRP3⁺ macrophages, and epithelial subsets, including AT2, AT1, ciliated, basal, club, and reactive epithelial cells. **B** Heatmaps showing the inferred communication roles of different cell populations within the TNF and IL-1 signaling pathway networks, including sender, receiver, mediator, and influencer scores. **C** Chord diagrams showing IL-1- and TNF-mediated intercellular communication patterns among epithelial and macrophage subpopulations. **D** Chord diagrams comparing TNF and IL-1 signaling networks between sepsis survivors and sepsis non-survivors. **E**, **F** Mitochondrial membrane potential in WT AT2 cells was assessed by JC-1 staining after co-culture with sham AMs or CLP AMs treated with IgG control, anti-TNF-α, anti-IL-1β, or combined anti-TNF-α and anti-IL-1β neutralizing antibodies, and quantified as the percentage of cells with low mitochondrial membrane potential. **G**, **H** Annexin V-FITC/PI staining was used to detect apoptosis in WT AT2 cells after co-culture with sham AMs or CLP AMs treated with IgG control, anti-TNF-α, anti-IL-1β, or combined anti-TNF-α and anti-IL-1β neutralizing antibodies, and the percentage of Annexin V⁺/PI⁺ cells was quantified. **I**, **J** Mitochondrial reactive oxygen species levels in WT AT2 cells were measured by flow cytometry after co-culture with sham AMs or CLP AMs in the presence of IgG control, anti-TNF-α, anti-IL-1β, or combined anti-TNF-α and anti-IL-1β neutralizing antibodies, and quantified as mean fluorescence intensity. **K** Western blot analysis of epithelial differentiation and barrier-related proteins in WT AT2 cells, including SP-C, E-cadherin, Occludin, TTF-1, and ZO-1, after co-culture with sham AMs or CLP AMs treated with IgG control or combined anti-TNF-α and anti-IL-1β neutralizing antibodies. GAPDH was used as the loading control. Data are presented as mean ± SEM. ^*^P < 0.05, ^**^P < 0.01, ^***^P < 0.001 versus Sham AM + IgG; ^#^*P* < 0.05, ^##^*P* < 0.01, ^###^*P* < 0.001 versus CLP AM + IgG. SA sepsis alive, SD sepsis dead, AM alveolar macrophage, AT2 alveolar epithelial type II cell, CLP cecal ligation and puncture, TNF-α tumor necrosis factor-α, IL-1β interleukin-1β, PI propidium iodide, ROS reactive oxygen species, MFI mean fluorescence intensity. Data are presented as mean ± SEM. ^*^P < 0.05, ^**^P < 0.01, ^***^P < 0.001.

To elucidate how pyroptotic macrophages influence epithelial injury, we performed cell-cell communication analysis across macrophage and epithelial subpopulations. Network analysis revealed extensive intercellular interactions, with a large number and high strength of connections observed between NLRP3⁺ macrophages and AT2 epithelial cells (Fig. 4A). Ligand–receptor analysis further identified TNF, IL-1, and CXCL family signals as major macrophage-derived pathways targeting AT2 epithelial cells, with NLRP3⁺ macrophages showing prominent communication probability toward AT2 cells (Fig. S1A).

We next focused on TNF and IL-1 signaling, two inflammatory pathways closely related to macrophage activation and epithelial injury. Heatmap analysis of communication roles showed that macrophage subsets, particularly NLRP3⁺ macrophages, exhibited strong outgoing signaling activity within the TNF and IL-1 networks, whereas epithelial populations, especially AT2 cells, showed prominent incoming signaling activity (Fig. 4B). Chord diagrams further demonstrated IL-1- and TNF-mediated communication between macrophage and epithelial subpopulations (Fig. 4C). Comparison between sepsis survivors and non-survivors showed enhanced TNF and IL-1 signaling networks in non-survivors, suggesting amplification of macrophage–epithelial inflammatory communication during fatal sepsis progression (Fig. 4D). Consistently, IL1B and TNF expression was enriched in NLRP3⁺ macrophages and were further increased in non-survivors compared with survivors (Fig. S1B, C).

To functionally validate whether macrophage-derived TNF and IL-1 signals mediate epithelial injury, WT AT2 cells were co-cultured with sham AMs or CLP AMs in the presence of IgG control, anti-TNF-α, anti-IL-1β, or combined anti-TNF-α and anti-IL-1β neutralizing antibodies. JC-1 staining showed that CLP AMs increased the proportion of AT2 cells with low mitochondrial membrane potential, whereas TNF-α and/or IL-1β neutralization partially restored mitochondrial membrane potential (Fig. 4E, F). Annexin V-FITC/PI staining showed increased apoptosis in AT2 cells after co-culture with CLP AMs, which was attenuated by TNF-α and/or IL-1β blockade (Fig. 4G, H). In parallel, mitochondrial ROS levels were increased in AT2 cells exposed to CLP AMs and were reduced after neutralization of TNF-α and/or IL-1β (Fig. 4I, J). Western blot analysis further showed that co-culture with CLP AMs reduced the expression of epithelial differentiation and barrier-related proteins in AT2 cells, including SP-C, E-cadherin, Occludin, TTF-1, and ZO-1, whereas combined TNF-α and IL-1β neutralization partially preserved their expression (Fig. 4K).

Together, these findings indicate that pyroptotic AM-derived TNF/IL-1 signaling contributes to AT2 mitochondrial dysfunction, oxidative stress, apoptosis, and epithelial barrier injury during sepsis-induced ALI.

### Zbp1 deficiency dampens inflammatory and pyroptotic signatures across lung cell types during sepsis, with a pronounced effect in myeloid populations

**Fig. 5:**
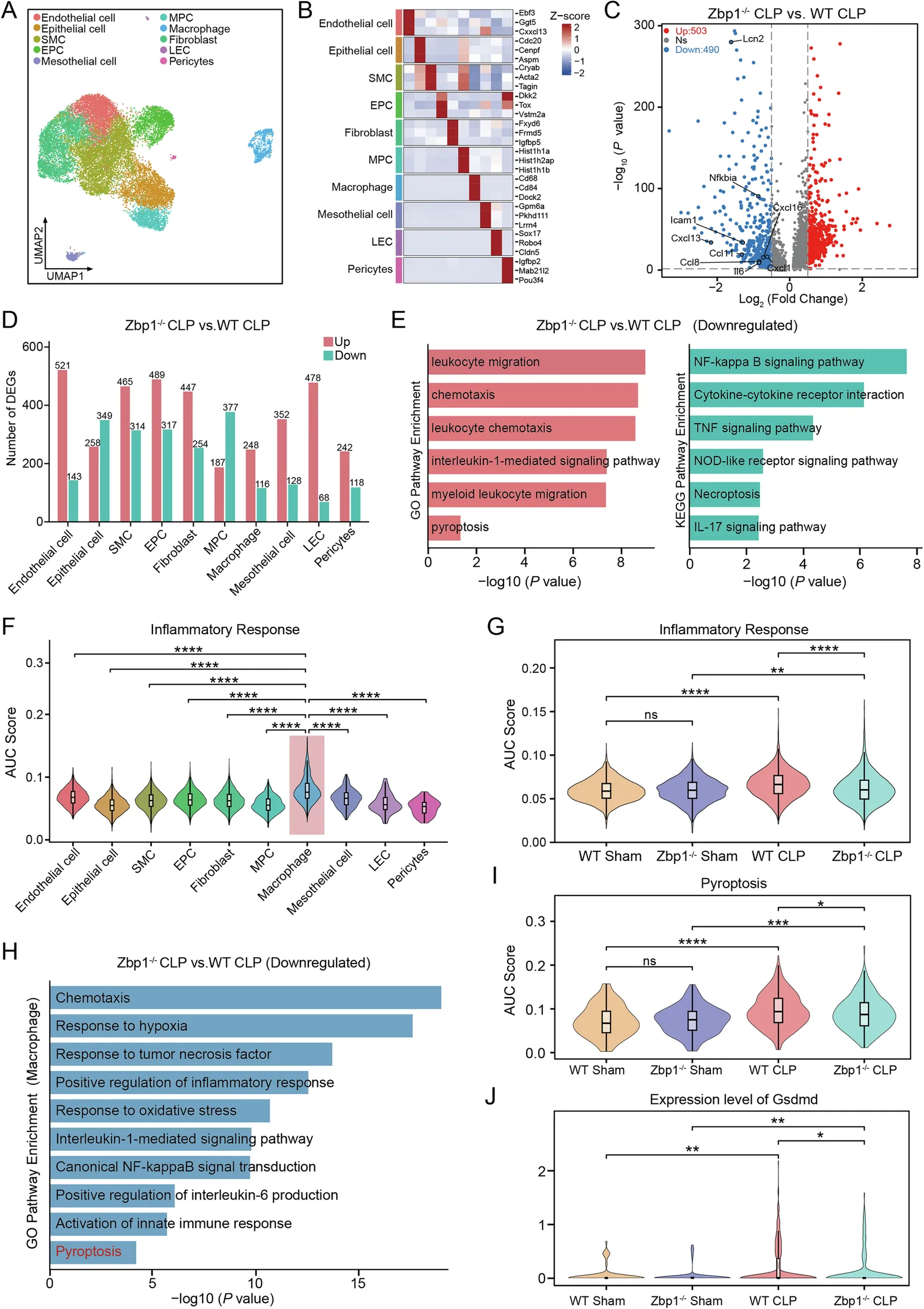
Single-cell validation reveals that *Zbp1* deficiency attenuates macrophage inflammatory activation and pyroptosis-related signaling during sepsis-induced ALI. Experimental design for mouse single-cell validation. Lung tissues from WT Sham (*n* = 3), WT CLP (*n* = 4), *Zbp1*^*−/−*^ Sham (*n* = 4), and *Zbp1*^*−/−*^ CLP (*n* = 3) were dissociated to single cells, profiled by 10x Genomics, and subjected to integrated analysis. **A** UMAP of lung cells annotated into endothelial cells, epithelial cells, smooth muscle cells (SMC), erythroid progenitors (EPC), fibroblasts, mononuclear phagocytes (MPC), macrophages, mesothelial cells, lymphatic endothelial cells (LEC), and pericytes. **B** Dot-plot of canonical markers confirming cluster identities across major compartments. **C** Volcano plot of *Zbp1*^*−/−*^ CLP vs WT CLP showing widespread differential expression with many inflammation/innate-immunity genes reduced in Zbp1 deficiency (representative genes labeled). **D** Numbers of DEGs per cell type between *Zbp1*^*−/−*^ CLP and WT CLP. **E** Pathway enrichment of downregulated genes in *Zbp1*^*−/−*^ CLP versus WT CLP: GO terms (left panel) include leukocyte migration, chemotaxis, IL-1–mediated signaling, myeloid leukocyte migration, pyroptosis, KEGG terms (right panel) include NF-κB, cytokine–cytokine receptor interaction, TNF, NOD-like receptor, necroptosis, IL-17 signaling. **F** Inflammatory-response scores across cell types (all CLP samples), indicating highest activity in macrophage. **G** Group comparison of inflammatory-response scores showing increase in WT CLP versus sham and reduction in *Zbp1*^*−/−*^ CLP. **H** GO enrichment of downregulated genes in macrophages (*Zbp1*^*−/−*^ CLP vs WT CLP) highlighting decreases in IL-1-mediated signaling, canonical NF-κB signaling, activation of innate immune response, and pyroptosis. **I** Group comparison of pyroptosis scores demonstrating elevation in WT CLP and attenuation in *Zbp1*^*−/−*^ CLP. **J** Expression of Gsdmd across groups showing induction in WT CLP and suppression with *Zbp1* deficiency. Data are presented as mean ± SEM. ^*^P < 0.05, ^**^P < 0.01, ^***^P < 0.001, ^****^P < 0.0001.

To validate the role of Zbp1 in vivo, we analyzed our previous single-cell RNA-seq datasets from CLP-induced sepsis in wild-type and *Zbp1*-deficient mice. Integrated clustering resolved the lung compartments (Fig. 5A) with markers confirming cell identities (Fig. 5B). Differential expression between *Zbp1*^*−/−*^ CLP and WT CLP revealed broad transcriptional shifts (Fig. 5C, D). Pathway analysis of downregulated genes in Zbp1-deficient lungs showed decreased activity of leukocyte migration and chemotaxis, IL-1-mediated signaling, and pyroptosis (GO), alongside reduced NF-κB, TNF, cytokine-receptor interaction, NOD-like receptor, necroptosis, and IL-17 pathways (KEGG) (Fig. 5E).

Macrophages exhibited a robust inflammatory response, which was elevated in WT CLP compared with sham and markedly attenuated in *Zbp1*^*−/−*^ CLP (Fig. 5F, G). Importantly, in macrophages, Zbp1 deficiency specifically downregulated IL-1-mediated signaling, canonical NF-κB signaling, activation of innate immune response, and pyroptosis (Fig. 5H). Concordantly, the pyroptosis score increased in WT CLP but declined with *Zbp1* knockout (Fig. 5I). Expression of Gsdmd, a key executioner of pyroptosis, was induced in WT CLP and suppressed in *Zbp1*^*−/−*^ CLP (Fig. 5J).

Collectively, these single-cell analyzes demonstrate that Zbp1 deletion attenuates inflammatory and pyroptotic programs in the septic lung, with prominent effects in macrophage compartments, supporting the central role of ZBP1 in driving macrophage-centric inflammatory injury observed in human datasets.

### ZBP1 promotes macrophage pyroptosis through interaction with NLRP3 during sepsis-induced ALI

**Fig. 6:**
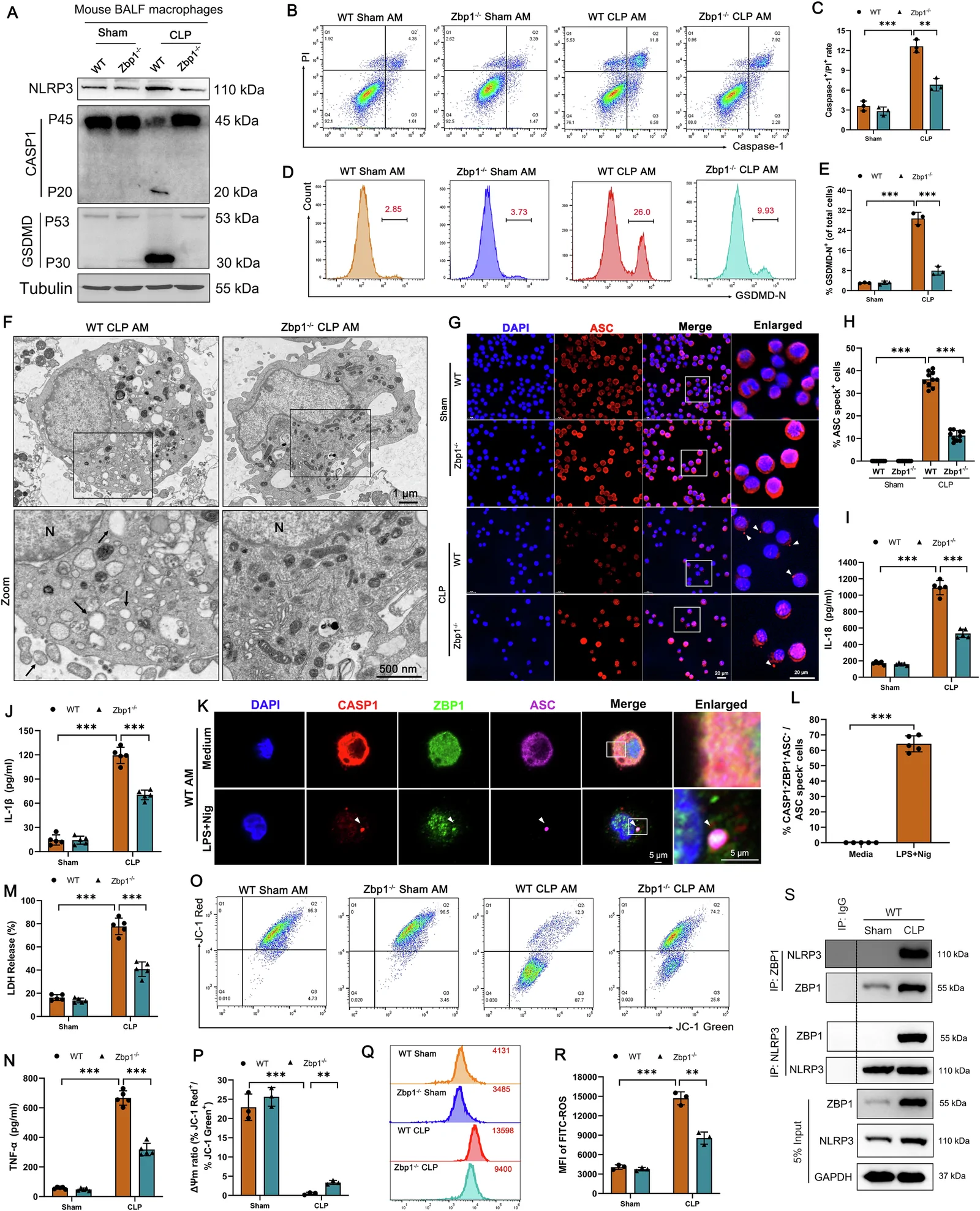
ZBP1 promotes macrophage pyroptosis and interacts with NLRP3 during sepsis-induced ALI. **A** Western blot analysis of BALF-derived alveolar macrophages (AMs) from WT and *Zbp1*^*−/−*^ mice under Sham or CLP conditions, showing the expression of NLRP3, cleavage of Caspase-1 (p20), and cleavage of GSDMD (p30/p53). **B** Flow cytometry plots displaying the proportion of active Caspase-1^+^ and PI^+^ double-positive macrophages. Cells were stained using a fluorescent-labeled caspase inhibitor probe (FAM-FLICA Caspase-1 Assay Kit) to detect active Caspase-1, followed by propidium iodide (PI) staining, and analyzed by flow cytometry. **C** Quantification of active Caspase-1⁺/PI⁺ macrophages (*n* = 3). **D** Flow cytometry analysis of GSDMD-N⁺ macrophages. **E** Quantification of GSDMD-N⁺ macrophages (*n* = 3). **F** Transmission electron microscopy (TEM) images showing ultrastructural changes in macrophages. WT CLP macrophages exhibit pyroptotic morphology, including cell swelling, membrane pore formation, and vacuole-like pyroptotic bodies (indicated by arrows), whereas *Zbp1*^*−/−*^ CLP macrophages display preserved cellular and mitochondrial structure. **G** Immunofluorescence staining of ASC specks in AMs. **H** Quantification of ASC speck⁺ macrophages. **I**, **J** ELISA measurement of IL-18 and IL-1β levels in BALF or macrophage supernatant (*n* = 5). **K** Confocal imaging showing colocalization of CASP1 (red), ZBP1 (green), and ASC (magenta) in macrophages after LPS + Nigericin stimulation. **L** Percentage of CASP1⁺ZBP1⁺ASC⁺ triple-positive macrophages. **M** LDH release assay showing membrane rupture in macrophages (*n* = 5). **N** ELISA measurement of TNF-α secretion (*n* = 5). **O** JC-1 staining of mitochondrial membrane potential (ΔΨm). WT CLP macrophages show increased green fluorescence (depolarized mitochondria), whereas *Zbp1*^*−/−*^ CLP macrophages preserve red fluorescence (intact ΔΨm). **P** Quantification of ΔΨm loss (red/green ratio). **Q**, **R** Intracellular ROS production measured by flow cytometry and quantified as MFI. **S** Co-immunoprecipitation analysis of the interaction between ZBP1 and NLRP3 in BALF-derived alveolar macrophages from WT Sham and WT CLP mice. Five percent of total lysate was used as input, and GAPDH served as the input loading control. Data are presented as the mean ± SD, ^*^P < 0.05, ^**^P < 0.01, ^***^P < 0.001.

To determine whether ZBP1 drives macrophage pyroptosis during sepsis-induced ALI, we isolated BALF-derived AMs from WT and *Zbp1*^*−/−*^ mice subjected to Sham or CLP. The absence of ZBP1 protein expression in BALF-derived AMs from *Zbp1*^*−/−*^ mice was confirmed by western blot analysis (Fig. S2A). Western blot analysis revealed that NLRP3 expression, Caspase-1 cleavage (p20), and GSDMD cleavage (p30/p53) were markedly induced in WT CLP, whereas *Zbp1*^*−/−*^ CLP macrophages displayed substantially reduced activation of all key pyroptosis-related proteins (Fig. 6A). Consistently, flow cytometry showed a significant increase in active Caspase-1⁺/PI⁺ double-positive macrophages in WT CLP mice, which was largely attenuated in *Zbp1*^*−/−*^ CLP mice (Fig. 6B, C). In parallel, GSDMD-N⁺ macrophages, indicating formation of membrane pores, were elevated in WT CLP and significantly reduced by *Zbp1* deficiency (Fig. 6D, E).

To further examine pyroptotic morphology, transmission electron microscopy demonstrated that WT CLP AMs exhibited typical pyroptotic ultrastructural features, including cell swelling, membrane pore formation, and vacuole-like pyroptotic bodies (arrow), whereas *Zbp1*^*−/−*^ CLP AMs preserved intact membrane and mitochondrial structure (Fig. 6F). Quantitative analysis further showed fewer pyroptotic bodies and more preserved mitochondria in *Zbp1*^*−/−*^ CLP AMs compared with WT CLP AMs (Fig. S2B). In line with these observations, ASC speck formation, a hallmark of inflammasome assembly, was strongly induced in WT CLP and significantly decreased in *Zbp1*^*−/−*^ CLP (Fig. 6G, H). Moreover, IL-18 and IL-1β secretion was markedly elevated in WT CLP and attenuated in *Zbp1*-deficient macrophages (Fig. 6I, J).

Mechanistically, confocal microscopy revealed colocalization of ZBP1 with CASP1 and ASC in LPS+Nigericin-treated macrophages, forming CASP1⁺ZBP1⁺ASC⁺ triple-positive inflammasome complexes, whereas such complexes were rarely detected under control conditions (Fig. 6K, L). Functionally, LDH release, reflecting plasma membrane rupture, was significantly increased in WT CLP but reduced in *Zbp1*^*−/−*^ CLP (Fig. 6M), and TNF-α production followed a similar pattern (Fig. 6N).

Because pyroptosis is tightly linked to mitochondrial stress, we next assessed oxidative damage. JC-1 staining revealed profound mitochondrial membrane potential (ΔΨm) loss in WT CLP macrophages, whereas *Zbp1*^*−/−*^ CLP macrophages preserved ΔΨm (Fig. 6O, P). Furthermore, ROS levels were markedly elevated in WT CLP AMs and significantly decreased in *Zbp1*^*−/−*^ CLP AMs (Fig. 6Q, R).

To further explore the mechanism linking ZBP1 to NLRP3 inflammasome activation, we examined the spatial association and interaction between ZBP1 and NLRP3. Co-immunoprecipitation analysis demonstrated an interaction between ZBP1 and NLRP3 in BALF-derived AMs from WT CLP mice (Fig. 6S). This interaction was further supported by proximity ligation assay, which showed increased ZBP1–NLRP3 proximity signals in CLP AMs compared with Sham AMs (Fig. S2C). Immunofluorescence staining and line-scan colocalization analysis also showed enhanced spatial overlap between ZBP1 and NLRP3 in CLP AMs (Fig. S2D). Together, these findings support a ZBP1–NLRP3-associated mechanism underlying macrophage pyroptosis during sepsis-induced ALI.

Collectively, these findings indicate that ZBP1 promotes NLRP3 inflammasome activation, CASP1 cleavage, GSDMD-mediated membrane pore formation, cytokine release, oxidative stress, mitochondrial dysfunction, and pyroptotic cell death in alveolar macrophages during sepsis-induced ALI. Further mechanistic evidence supports the involvement of ZBP1–NLRP3 interaction in macrophage pyroptosis, consistent with our single-cell and intercellular communication analyzes.

### Pyroptotic macrophages compromise epithelial barrier integrity and disrupt mitochondrial homeostasis in AT2 cells via ZBP1-dependent signaling

**Fig. 7:**
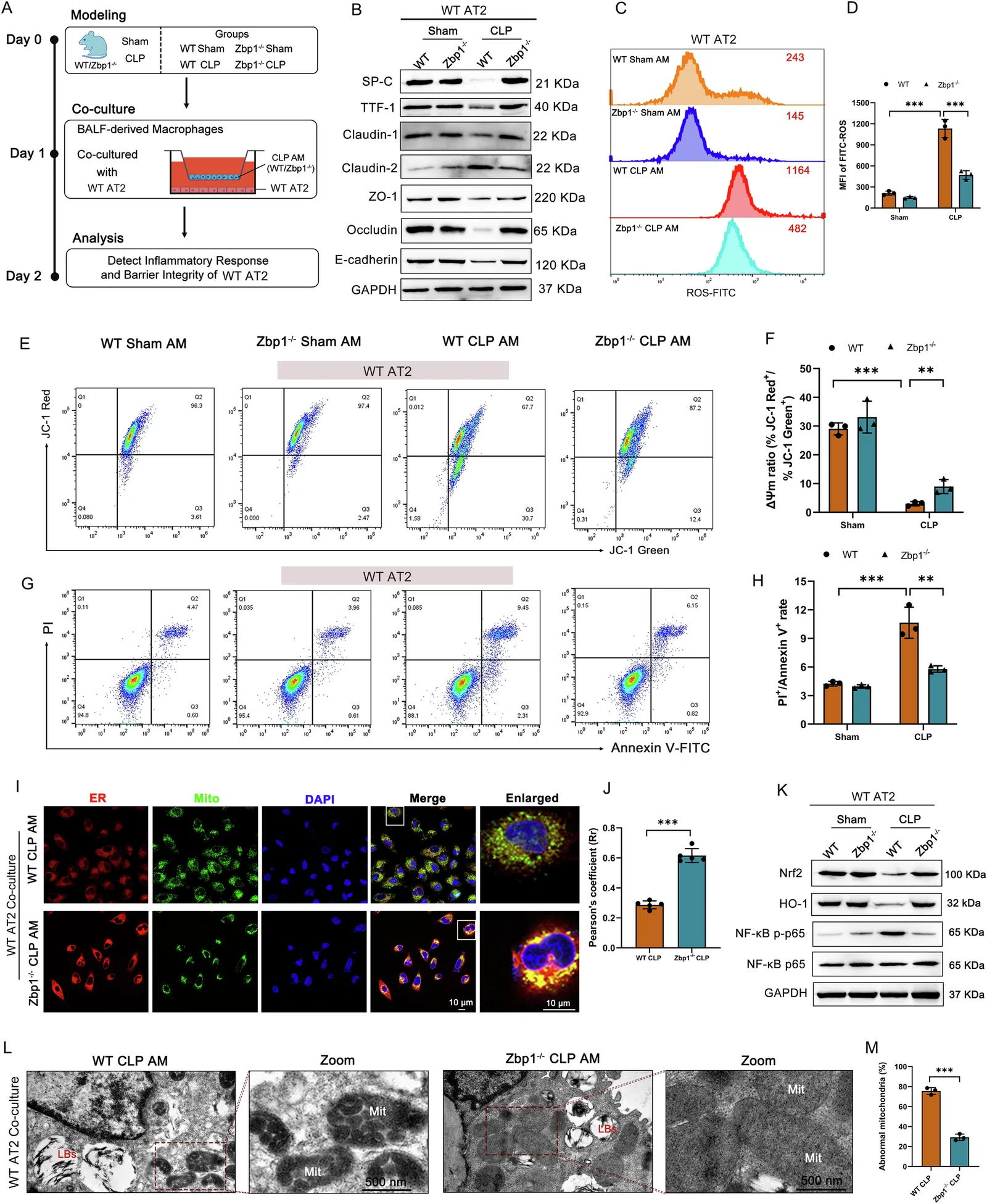
Pyroptotic macrophages induce mitochondrial damage and epithelial dysfunction in alveolar type II cells through ZBP1-dependent signaling. **A** Schematic of the co-culture experiment. BALF-derived macrophages from WT or *Zbp1*^*−/−*^ mice under Sham or CLP conditions were co-cultured with WT alveolar type II (AT2) cells for 24 h prior to analysis. **B** Western blot was performed to assess epithelial differentiation and barrier-related proteins in AT2, including SP-C, TTF-1, Claudin-1, Claudin-2, ZO-1, Occludin, and E-cadherin. **C**, **D** Intracellular reactive oxygen species (ROS) levels in AT2 were measured by flow cytometry using FITC-labeled ROS probes and quantified as mean fluorescence intensity. **E**, **F** Mitochondrial membrane potential (ΔΨm) in AT2 was evaluated by JC-1 staining and analyzed by flow cytometry, and the red/green fluorescence ratio was calculated. **G**, **H** Apoptosis and necrosis of AT2 were detected by Annexin V and propidium iodide (PI) staining using flow cytometry, and the percentage of PI⁺/Annexin V⁺ cells was quantified. **I**, **J** Confocal microscopy was used to visualize the endoplasmic reticulum (ER) and mitochondria in AT2, and their spatial colocalization was quantified by Pearson’s correlation coefficient. **K** Western blot analysis of oxidative stress and inflammatory signaling in AT2, including Nrf2, HO-1, and NF-κB p65. **L** Transmission electron microscopy (TEM) was used to examine ultrastructural features of AT2 cells, including mitochondrial morphology and lamellar body structure. **M** Quantitative analysis of the percentage of abnormal mitochondria in AT2 cells was performed based on the TEM images. Data are presented as the mean ± SD, ^*^P < 0.05, ^**^P < 0.01, ^***^P < 0.001.

To investigate whether pyroptotic macrophages impair alveolar epithelial function, BALF-derived macrophages from WT or *Zbp1*^*−/−*^ mice subjected to Sham or CLP were co-cultured with WT AT2 cells for 24 h (Fig. 7A). AT2 epithelial phenotype and barrier integrity were examined. Western blot analysis showed that co-culture with WT CLP macrophages reduced epithelial differentiation markers (SP-C, TTF-1) and tight-junction/adhesion proteins (ZO-1, Occludin, E-cadherin), decreased Claudin-1 and increased Claudin-2 in AT2, whereas exposure to *Zbp1*^*−/−*^ CLP macrophages largely mitigated these alterations (Fig. 7B), suggesting that ZBP1-dependent macrophage pyroptosis contributes to epithelial barrier disruption.

We next evaluated oxidative stress and mitochondrial function in AT2 cells. Flow cytometry revealed a pronounced increase in ROS generation in AT2 cells exposed to WT CLP macrophages, while Zbp1 deficiency significantly reduced ROS levels (Fig. 7C, D). Consistently, JC-1 staining demonstrated a substantial loss of mitochondrial membrane potential (ΔΨm) in AT2 cells co-cultured with WT CLP macrophages, whereas *Zbp1*^*−/−*^ CLP macrophages preserved mitochondrial integrity (Fig. 7E, F). Moreover, Annexin V/PI staining showed enhanced AT2 cell death following co-culture with WT CLP macrophages, whereas *Zbp1* deficiency markedly attenuated apoptosis and necrosis (Fig. 7G, H).

To further characterize mitochondrial stress, confocal imaging revealed that co-culture with WT CLP macrophages reduced endoplasmic reticulum (ER)-mitochondria colocalization in AT2, indicative of disrupted mitochondria-associated endoplasmic reticulum membrane (MAM) integrity, whereas *Zbp1*^*−/−*^ CLP macrophages preserved ER-mitochondria interactions, as reflected by a higher Pearson’s coefficient (Fig. 7I, J). At the signaling level, WT CLP macrophages activated NF-κB and suppressed Nrf2/HO-1 antioxidant pathways in AT2 cells, whereas *Zbp1* deficiency reversed these effects, indicating restoration of redox balance (Fig. 7K). Finally, transmission electron microscopy confirmed that WT CLP macrophages induced severe mitochondrial swelling, cristae fragmentation, and lamellar body loss in AT2 cells, while Zbp1-deficient macrophages preserved ultrastructural integrity (Fig. 7L, M).

Together, these findings indicate that ZBP1-dependent macrophage pyroptosis contributes to increased oxidative stress, mitochondrial dysfunction, epithelial barrier impairment, and cell death in AT2 cells, supporting a functional association between macrophage pyroptosis and epithelial injury in sepsis-induced ALI.

### *Zbp1* deficiency disrupts macrophage-AT2 crosstalk and reduces pathogenic intercellular signaling in septic lungs

**Fig. 8:**
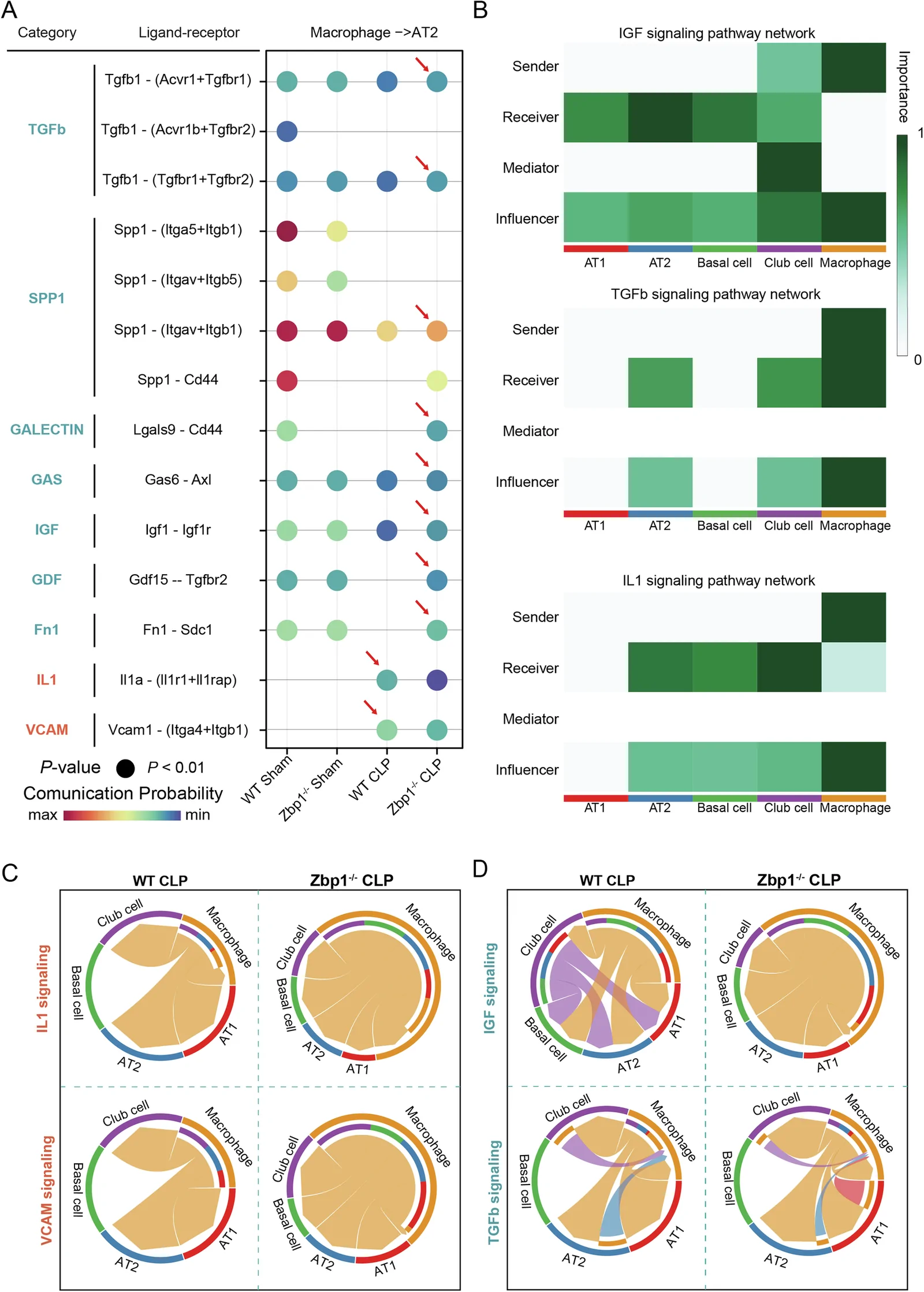
Zbp1 deficiency alters macrophage–AT2 intercellular communication networks in septic lungs. **A** Dot plot of predicted ligand–receptor interactions from macrophages to AT2 cells across experimental groups (WT Sham, *Zbp1*^*−/−*^ Sham, WT CLP, *Zbp1*^*−/−*^ CLP). Ligand-receptor pairs were categorized into inflammatory, profibrotic, growth factor, and adhesion-related signaling. Dot size indicates statistical significance (*P* < 0.01), and color represents communication probability. **B** Heatmap showing the identification of signaling roles for each cell population (macrophage, AT2, AT1, basal cell, club cell) as sender, receiver, mediator, or influencer within representative signaling pathways. **C**, **D** Chord plots showing intercellular communication networks among macrophages and epithelial subtypes (AT2, AT1, basal, and club cells) in WT CLP and *Zbp1*^*−/−*^ CLP lungs. Representative pathways involved in cell-cell crosstalk, including IGF, TGFβ, IL-1, and VCAM signaling, were inferred using the CellChat R package.

To determine whether ZBP1 shapes intercellular communication between macrophages and alveolar epithelial cells, we performed cell-cell interaction analysis using lung single-cell transcriptomes from WT and *Zbp1*^*−/−*^ mice after CLP. We first identified major epithelial subtypes, including AT2, AT1, club, and basal cells, based on canonical marker gene expression (Fig. S3A, B). AT2 cells displayed relatively enriched inflammatory and adhesion-related transcriptional features, supporting their selection for subsequent macrophage–AT2 communication analysis (Fig. S3C). To characterize the macrophage-epithelial crosstalk, we examined ligand-receptor interactions specifically from macrophages to AT2 cells. Predicted interactions involved inflammatory cytokines, profibrotic factors, growth factors, and adhesion molecules. *Zbp1* deficiency enhanced reparative signaling, such as TGF and IGF signaling, while reducing injury-associated inflammatory cues like IL1 signaling in macrophage-epithelial communication (Fig. 8A).

For these signaling pathways, network role analysis revealed that in WT CLP lungs, macrophages acted as dominant senders and influencers within the signaling network, whereas AT2 cells were the primary receivers, indicating a unidirectional and hierarchical communication axis from macrophages to epithelial cells (Fig. 8B).

Network visualization revealed extensive communication between macrophages and epithelial populations in WT CLP lungs, with AT2 cells receiving substantial input from macrophages across multiple signaling axes (Fig. 8C, D). In contrast, the overall connectivity between macrophages and AT2 cells was markedly altered in *Zbp1*^*−/−*^ CLP lungs, suggesting that Zbp1 deficiency remodels the intercellular signaling landscape.

Together, these findings indicate that ZBP1 is required to maintain the pathogenic crosstalk from macrophages to AT2 epithelial cells during sepsis-induced ALI. By modulating intercellular communication strength and network hierarchy, ZBP1-dependent macrophage signaling serves as a central mechanism linking innate immune activation to epithelial dysfunction in the septic lung.

## Discussion

In this study, we observed the expansion of pyroptosis-related alveolar macrophage subsets during sepsis-induced ALI and found that ZBP1 interacted with NLRP3 to promote macrophage pyroptosis. ZBP1-associated macrophage pyroptosis increased the release of inflammatory mediators, particularly IL-1β and TNF-α, which enhanced macrophage–AT2 inflammatory crosstalk and contributed to AT2 epithelial injury. Together, these findings suggest that the ZBP1–NLRP3 inflammasome axis links macrophage pyroptosis to cytokine-mediated epithelial injury during sepsis-induced ALI.

Macrophages are key immune sentinels within the pulmonary microenvironment, orchestrating inflammation, tissue repair, and host defense [22, 23]. However, the conventional M1/M2 polarization framework has proven insufficient to capture the true heterogeneity and functional diversity of lung macrophages [24]. In this study, single-cell transcriptomic analysis revealed marked macrophage heterogeneity during sepsis-induced ALI. Among the identified macrophage subsets, NLRP3⁺ macrophages displayed enrichment of pyroptosis-related genes, including IL1B, NLRP3, CASP1, and ZBP1, supporting their designation as pyroptosis-associated macrophages [25, 26]. Recent advances in single-cell sequencing have enabled finer delineation of macrophage heterogeneity, uncovering distinct subpopulations with specialized roles in inflammation and tissue remodeling [27, 28]. These findings suggest that pyroptosis-associated macrophages may represent an inflammatory macrophage state that is more closely linked to epithelial injury than conventional polarization categories during sepsis-induced ALI.

Our results revealed markedly increased expression and activation of ZBP1 in macrophages during sepsis. Exogenous pathogen-derived nucleic acids, together with endogenous damage-associated molecular patterns exemplified by mitochondrial DNA (mtDNA), synergistically initiate and sustain ZBP1 activation in septic pathology [5, 20, 29]. ZBP1 functions as an innate immune receptor that senses nucleic acids and activates inflammatory signaling [16]. ZBP1 has also been recognized as an upstream regulator of programmed cell death and pro-inflammatory pathways [13, 29]. In the present study, we detected ZBP1–NLRP3 interaction in macrophages, accompanied by inflammasome assembly, caspase-1 activation, and pyroptosis. While previous studies have demonstrated that ZBP1 senses influenza A virus proteins to trigger inflammatory responses and NLRP3 inflammasome activation via the RIPK1-RIPK3-caspase-8 axis during viral infection [18, 30], our results extend this regulatory paradigm to septic conditions. Notably, emerging evidence has demonstrated that mitochondrial ZBP1 facilitates mitochondrial recruitment of NLRP3 through MAVS to drive inflammasome activation [31, 32]. Our findings further reveal that ZBP1-NLRP3 interaction contributes to macrophage pyroptosis in sepsis-induced ALI. Consistently, *Zbp1* deficiency attenuated inflammatory activation and pyroptotic responses in alveolar macrophages, accompanied by reduced inflammasome activation and decreased release of inflammatory cytokines, including TNF-α and IL-1β. These findings suggest that ZBP1-associated inflammasome activation may promote macrophage-derived inflammatory mediator release, thereby contributing to macrophage-AT2 inflammatory crosstalk during sepsis-induced ALI.

In the present study, ligand–receptor analysis revealed that pyroptosis-associated macrophages preferentially communicated with AT2 cells through pro-inflammatory pathways, particularly TNF and IL-1 family signaling. This inflammatory communication pattern was enhanced in non-survivors, indicating that macrophage-derived TNF/IL-1 signals may represent an important macrophage-epithelial interaction hub associated with disease progression [33]. Functionally, cytokine neutralization experiments further supported this prediction, as blockade of TNF-α and/or IL-1β partially attenuated CLP macrophage-induced mitochondrial dysfunction, apoptosis, and barrier-related protein loss in AT2 cells. These findings suggest that TNF and IL-1 signaling provide a functional link between ZBP1-associated macrophage pyroptosis and AT2 epithelial dysfunction. In mouse models, Zbp1 deficiency was accompanied by reduced pro-inflammatory macrophage–AT2 communication and a shift toward alternative signaling programs, including pathways previously associated with tissue remodeling or immune regulation [34, 35]. Together, these results suggest that TNF-α/IL-1β-mediated macrophage–AT2 inflammatory crosstalk represents an important link between ZBP1-associated macrophage pyroptosis and epithelial injury during sepsis-induced ALI.

Co-culture experiments indicated that ZBP1-associated macrophage activation contributes to AT2 epithelial dysfunction during sepsis-induced ALI. Macrophage-derived inflammatory signals were associated with impaired epithelial differentiation and barrier integrity in AT2 cells, together with increased oxidative stress and mitochondrial dysfunction. MAMs are essential contact sites that coordinate calcium, lipid, and redox exchange between the endoplasmic reticulum and mitochondria [36], and MAM disruption has been linked to oxidative stress, mitochondrial fragmentation, and cell death under inflammatory conditions [37, 38]. Consistently, activation of NF-κB signaling together with suppression of the Nrf2/HO-1 antioxidant pathway further indicated an imbalance between inflammatory activation and antioxidant defense in AT2 cells. These findings suggest that ZBP1-associated macrophage pyroptosis may impair AT2 epithelial homeostasis by promoting inflammatory signaling, barrier disruption, mitochondrial stress, and altered organelle communication during sepsis-induced ALI.

These findings may have potential value for understanding macrophage-driven inflammatory injury during sepsis-induced ALI. Excessive macrophage pyroptosis amplifies pulmonary inflammation and accelerates alveolar barrier failure [39, 40]. Nevertheless, several limitations should be noted. First, although this study integrated human BALF single-cell data with mouse models and cell-based validation, functional validation using human primary alveolar macrophages or human AT2 epithelial cells was not performed. Second, although Zbp1 deficiency reduced macrophage pyroptosis and epithelial injury, ZBP1 overexpression rescue or selective pharmacological inhibition was not investigated in the current study. Third, the single-cell RNA-seq analyzes were based on publicly available datasets, and potential dataset-related variability should be considered. Future studies using human primary cell systems, optimized ZBP1-targeted intervention strategies, and spatially resolved approaches may further clarify ZBP1-associated macrophage-epithelial interactions during sepsis-induced ALI.

In conclusion, this study identifies pyroptosis-related alveolar macrophage subsets that expand during sepsis-induced ALI. Our findings suggest that ZBP1 interacts with NLRP3 to promote macrophage inflammasome activation, pyroptosis, and inflammatory mediator release. These macrophage-derived signals, particularly IL-1β and TNF-α, enhance macrophage–AT2 inflammatory crosstalk and contribute to epithelial mitochondrial dysfunction and barrier impairment. Together, these results support a mechanism linking ZBP1-associated macrophage pyroptosis to AT2 epithelial injury during sepsis-induced ALI and provide a basis for further investigation of ZBP1-associated macrophage–epithelial interactions.

## Materials and methods

### Participant recruitment and sample collection

This study was approved by the Institutional Ethics Committee of Shenzhen Hospital, Southern Medical University (NYSZYYEC2025K084R002). Informed consent was obtained from all subjects (or their legally authorized representatives) prior to their participation in the study. All study procedures were conducted in compliance with relevant ethical guidelines and regulations. Healthy volunteers were recruited through hospital personnel notices. Critically ill patients admitted to the intensive care unit (ICU) of Shenzhen Hospital between January 2019 and December 2023 were screened. The diagnosis of sepsis and septic shock was based on the criteria defined by the Third International Consensus Definitions (Sepsis-3).

### Single-cell RNA sequencing datasets and analysis

Human BALF scRNA-seq datasets (GSE263469, GSM3660649, and GSE145926) were obtained from the Gene Expression Omnibus. Mouse scRNA-seq datasets (GSE278767), including our previously published CLP model data [20], were also downloaded. Data were processed in R using Seurat V5.0.1. Cells with mitochondrial or ribosomal gene content exceeding 10% of total expression, fewer than 200 or more than 3500 detected genes, or total UMI counts below 500 were also removed. UMI counts were normalized using the NormalizeData function (logNormalize, scale factor = 10,000). The FindVariableFeatures function was then used to identify the top 2000 highly variable genes. To mitigate batch effects among samples, we applied Harmony integration. Principal component analysis was performed based on the expression profiles of highly variable genes, and the top 30 principal components were selected for downstream analysis. Cell clustering was conducted using the FindNeighbors and FindClusters functions with optimized resolution parameters, and the RunUMAP function was used to visualize the clusters.

### Enrichment analysis

DEGs were identified between cell groups based on thresholds of *P* < 0.05 and |log₂FC| > 0.5. Pathway enrichment analysis of DEGs was performed using ClusterProfiler (v4.14.6). Gene set activity of pyroptosis (REACTOME_PYROPTOSIS) and inflammatory response (HALLMARK_INFLAMMATORY_RESPONSE) signatures in macrophages was performed using AUCell (v1.28.0), with gene sets obtained from MSigDB database.

### Pseudotime trajectory analysis

Macrophage trajectories were constructed using Monocle (v2.34.1). Genes with mean expression ≥0.1 were selected. Dimensionality reduction used DDRTree (max_components = 2, num_dim = 10). BEAM was used to identify state-associated genes (*q* < 0.01), followed by hierarchical clustering. ClusterGVis (v0.1.3) was used to define four clusters (num_clusters = 4).

### Cell–cell communication analysis

CellChat (v1.6.1) was used to evaluate intercellular communication changes between macrophages and alveolar epithelial cells.

### Animals

Male C57BL/6 and *Zbp1*^*−/−*^ mice (6–8 weeks) were purchased from Shanghai Model Organisms Center, Inc. (Shanghai, China). Mice were maintained under SPF conditions. Sample size was determined based on pre-experimental results and practices commonly used in similar animal and cell-based studies. Within the genotype, mice were allocated randomly into different experimental groups. Blinding was not performed for this study. All procedures complied with institutional animal welfare guidelines and the Guide for the Care and Use of Laboratory Animals. All animal experiments were performed in accordance with institutional guidelines and approved by the Ethics Committee on Laboratory Animal Care of Southern Medical University, Shenzhen Hospital (Approval No. 2024-0228).

### Cecal ligation and puncture (CLP) model

CLP was performed under isoflurane anesthesia. After midline laparotomy, the cecum was exteriorized, ligated with 4-0 silk, and punctured once using a 22-gauge needle. The abdomen was closed, and mice were sacrificed 24 h later for BALF and lung tissue collection.

### BALF macrophage isolation and culture

BALF was centrifuged at 300 × *g* for 10 min at 4 °C. Cell pellets were resuspended in DMEM containing 10% FBS and penicillin/streptomycin (50 μg/mL). After 2 h incubation, non-adherent cells were removed.

### Isolation and culture of primary AT2 cells

Primary AT2 cells were isolated from young C57BL/6 mice following standard procedures. Lung tissue was digested with collagenase and DNase I. After inhibition of trypsin activity and filtration, cells were incubated on IgG-coated plates to deplete non-adherent populations, and remaining AT2 cells were cultured in AT2-specific medium supplemented with antibiotics and FBS.

### Transmission electron microscopy

Cells were fixed in glutaraldehyde and washed with phosphate-buffered saline (PBS), dehydrated through an ethanol series, and post-fixed in osmium tetroxide. Samples were embedded in epoxy resin, polymerized overnight, sectioned, and stained with uranyl acetate and lead citrate. Ultrathin sections were examined using a Hitachi transmission electron microscope (TEM) system.

### Apoptosis assay

Apoptotic cells were detected using an Annexin V-FITC/PI Cell Apoptosis Detection Kit (CWBIO, China). After treatment, cells were collected, washed twice with cold PBS, and resuspended in binding buffer. Annexin V-FITC and propidium iodide were added according to the manufacturer’s instructions, followed by incubation in the dark at room temperature. Samples were analyzed immediately on a flow cytometer, and apoptosis rates were quantified using FlowJo software.

### Pyroptosis detection

Pyroptosis was assessed using a FAM-FLICA Caspase-1 Activity Assay Kit (MCE, China). Cells were incubated with the FAM-FLICA probe to label active Caspase-1, followed by PI staining according to the manufacturer’s instructions. Caspase-1⁺/PI⁺ cells were analyzed immediately by flow cytometry, and data were quantified using FlowJo software.

### Western blotting

Proteins were extracted using RIPA buffer with protease inhibitors (Sigma, USA). Protein concentrations were measured using the BCA assay (Thermo, USA). Equal protein (30 μg) was separated by 10% sodium dodecyl sulfate-polyacrylamide gel electrophoresis (SDS-PAGE) and transferred to PVDF membranes (Merck Millipore, Germany). Membranes were blocked in 5% milk for 1 h, incubated with primary antibodies overnight at 4 °C, then with HRP-conjugated secondary antibodies and visualized using ECL (Millipore). Primary antibodies included NLRP3, ZBP1, caspase-1 (pro-P45 / cleaved P20), GSDMD (P53 / P30), SP-C, TTF-1, Claudin-1, Claudin-2, ZO-1, Occludin, E-cadherin, Nrf2, HO-1, phospho-NF-κB p65, and total NF-κB p65. GAPDH or α-Tubulin served as loading controls. Full antibody information is provided in Supplementary Table S1. Blots were imaged with the ChemiDoc System (Bio-Rad, USA).

### ELISA

BALF macrophage culture supernatants (6 h, without additional stimulation) were analyzed for TNF-α, IL-18, and IL-1β using commercial ELISA kits (EIAab, Wuhan, China) according to the manufacturer’s instructions.

### Mitochondrial membrane potential assay

Mitochondrial membrane potential was evaluated using the JC-1 assay kit (Meilunbio, China). After treatment, cells were incubated with JC-1 working solution according to the manufacturer’s protocol, washed, and immediately analyzed using a flow cytometer. The proportion of JC-1 red/green fluorescence was quantified in FlowJo to assess changes in ΔΨm.

### Transwell co-culture

Primary WT AT2 cells (2 × 10⁴ cells/well) were seeded in the upper chamber of 0.4-µm Transwell inserts (Corning Costar) and grown to confluence. Macrophages (1 × 10⁵ cells/well) isolated from BALF of sham or CLP WT or *Zbp1*^*−/−*^ mice were added to the lower chamber. Co-cultures were maintained in complete DMEM at 37 °C, 5% CO₂ for 24 h. For cytokine neutralization experiments, anti-mouse IL-1β antibody (B122, eBioscience), anti-mouse TNF-α antibody (MP6-XT22, BioLegend), or a combination of both antibodies was added to the culture medium at a final concentration of 25 μg/mL for each antibody. The corresponding isotype controls, including Armenian hamster IgG isotype control (eBio299Arm, eBioscience) and LEAF™ purified rat IgG1, κ isotype control (RTK2071, BioLegend), were used alone or in combination, as appropriate. The antibodies were maintained throughout the co-culture period. AT2 cells were then harvested for downstream assays. The neutralizing antibodies and corresponding isotype controls used for cytokine neutralization experiments are listed in Supplementary Table S2.

### Proximity ligation assay (PLA)

For PLA, macrophages were seeded in confocal dishes, washed with PBS, and fixed with 4% formaldehyde for 15 min. After blocking with 5% BSA for 30 min, cells were incubated overnight at 4 °C with primary antibodies against ZBP1 and NLRP3. Subsequent probe incubation, ligation, and amplification steps were performed according to the manufacturer’s instructions using the Duolink Detection Kit (DUO92102-1KT, Sigma-Aldrich, USA). Nuclei were counterstained with DAPI, and the samples were mounted for imaging. PLA signals were visualized and analyzed using a Nikon A1R confocal microscope with a 60× objective.

### Immunoprecipitation

For co-immunoprecipitation, macrophages were lysed in IP lysis buffer (Thermo Fisher Scientific, USA) on ice for 30 min. After centrifugation at 12,000 × *g* for 15 min at 4 °C, equal amounts of lysates were precleared with protein A/G magnetic beads (Merck Millipore, Germany) for 1 h at 4 °C. The precleared lysates were then incubated overnight at 4 °C with anti-ZBP1, anti-NLRP3, or control IgG antibodies. Protein A/G magnetic beads were added for an additional 2 h, followed by washing with cold PBS. Bound proteins were eluted in loading buffer, separated by SDS-PAGE, transferred to PVDF membranes, and analyzed by immunoblotting. For reciprocal co-immunoprecipitation, ZBP1 immunoprecipitates were blotted for NLRP3, and NLRP3 immunoprecipitates were blotted for ZBP1. Input lysates were used to confirm protein expression.

### Immunofluorescence

Cells or tissues were fixed in 4% paraformaldehyde for 30 min, permeabilized with 0.1% Triton X-100, and blocked with 5% BSA. Primary antibodies against ASC, ZBP1, caspase-1, etc. (Supplementary Table S1) were incubated overnight at 4 °C. Fluorescent secondary antibodies were applied for 1 h in the dark, followed by DAPI nuclear staining. Imaging was performed using a Nikon A1R confocal microscope.

### Statistical analysis

Data were analyzed using R Studio (v4.4.1) and GraphPad Prism (v9.5). Results are presented as mean ± SD from ≥3 independent experiments or median interquartile range (IQR) where appropriate. Student’s *t*-test or one-way analysis of variance (ANOVA) was used as indicated. *P* < 0.05 was considered statistically significant.

## Supplementary information

Supplementary Figures

Supplementary Tables

Full uncropped western blots

## Data Availability

The datasets supporting the findings of this study are available from the corresponding author upon reasonable request. No restrictions apply to data access.

## References

[1] Singer M, Deutschman CS, Seymour CW, Shankar-Hari M, Annane D, Bauer M, et al. The Third International Consensus Definitions for Sepsis and Septic Shock (Sepsis-3). JAMA. 2016;315:801–10. 26903338 10.1001/jama.2016.0287 PMC4968574

[2] Rudd KE, Johnson SC, Agesa KM, Shackelford KA, Tsoi D, Kievlan DR, et al. Global, regional, and national sepsis incidence and mortality, 1990-2017: analysis for the Global Burden of Disease Study. Lancet. 2020;395:200–11. 31954465 10.1016/S0140-6736(19)32989-7 PMC6970225

[3] Genga KR, Russell JA. Update of Sepsis in the Intensive Care Unit. J Innate Immun. 2017;9:441–55. 28697503 10.1159/000477419 PMC6738902

[4] Gong T, Zhang XD, Peng ZY, Ye YF, Liu RM, Yang YG, et al. Macrophage-derived exosomal aminopeptidase N aggravates sepsis-induced acute lung injury by regulating necroptosis of lung epithelial cell. Commun Biol. 2022;5:543. 35668098 10.1038/s42003-022-03481-y PMC9170685

[5] Gong T, Wang QD, Loughran PA, Li YH, Scott MJ, Billiar TR, et al. Mechanism of lactic acidemia-promoted pulmonary endothelial cells death in sepsis: role for CIRP-ZBP1-PANoptosis pathway. Mil Med Res. 2024;11:71. 39465383 10.1186/s40779-024-00574-z PMC11514876

[6] Chen X, Wu R, Li L, Zeng YN, Chen JR, Wei MY, et al. Pregnancy-induced changes to the gut microbiota drive macrophage pyroptosis and exacerbate septic inflammation. Immunity. 2023;56:336–52. 36792573 10.1016/j.immuni.2023.01.015

[7] Wu CQ, Lu W, Zhang Y, Zhang GY, Shi XY, Hisada Y, et al. Inflammasome activation triggers blood clotting and host death through pyroptosis. Immunity. 2019;50:1401–11. 31076358 10.1016/j.immuni.2019.04.003 PMC6791531

[8] Lamkanfi M, Dixit VM. Mechanisms and functions of inflammasomes. Cell. 2014;157:1013–22. 24855941 10.1016/j.cell.2014.04.007

[9] Kim HM, Kim YM. HMGB1: LPS delivery vehicle for caspase-11-mediated pyroptosis. Immunity. 2018;49:582–4. 30332623 10.1016/j.immuni.2018.09.021

[10] Manda P, Feng YJ, Lyons JD, Berger SB, Otani S, DeLaney A, et al. Caspase-8 collaborates with caspase-11 to drive tissue damage and execution of endotoxic shock. Immunity. 2018;49:42–55. 30021146 10.1016/j.immuni.2018.06.011 PMC6064639

[11] Robinson KS, Toh GA, Rozario P, Chua R, Bauernfried S, Sun ZJ, et al. ZAKα-driven ribotoxic stress response activates the human NLRP1 inflammasome. Science. 2022;377:328–34. 35857590 10.1126/science.abl6324 PMC7614315

[12] Maelfait J, Rehwinkel J. The Z-nucleic acid sensor ZBP1 in health and disease. J Exp Med. 2023;220:e20221156. 37450010 10.1084/jem.20221156 PMC10347765

[13] Jiao HP, Wachsmuth L, Kumari S, Schwarzer R, Lin J, Eren RO, et al. Z-nucleic-acid sensing triggers ZBP1-dependent necroptosis and inflammation. Nature. 2020;580:391–5. 32296175 10.1038/s41586-020-2129-8 PMC7279955

[14] Rothenburg S, Schwartz T, Koch-Nolte F, Haag F. Complex regulation of the human gene for the Z-DNA binding protein DLM-1. Nucleic Acids Res. 2002;30:993–1000. 11842111 10.1093/nar/30.4.993 PMC100341

[15] Kuriakose T, Kanneganti TD. ZBP1: innate sensor regulating cell death and inflammation. Trends Immunol. 2018;39:123–34. 29236673 10.1016/j.it.2017.11.002 PMC5863909

[16] Yuan FF, Cai JZ, Wu JF, Tang YT, Zhao K, Liang F, et al. Z-DNA binding protein 1 promotes heatstroke-induced cell death. Science. 2022;376:609–15. 35511979 10.1126/science.abg5251

[17] Yin C, Fedorov A, Guo H, Crawford JC, Rousseau C, Zhong X, et al. Host cell Z-RNAs activate ZBP1 during virus infections. Nature. 2025;648:707–16. 41082924 10.1038/s41586-025-09705-5 PMC12711578

[18] Kuriakose T, Man SM, Malireddi RKS, Karki R, Kesavardhana S, Place DE, et al. ZBP1/DAI is an innate sensor of influenza virus triggering the NLRP3 inflammasome and programmed cell death pathways. Sci Immunol. 2016;1:aag2045. 27917412 10.1126/sciimmunol.aag2045 PMC5131924

[19] Yang DW, Liang YJ, Zhao SB, Ding Y, Zhuang QY, Shi QL, et al. ZBP1 mediates interferon-induced necroptosis. Cell Mol Immunol. 2020;17:356–68. 31076724 10.1038/s41423-019-0237-x PMC7109092

[20] Gong T, Fu Y, Wang QD, Loughran PA, Li YH, Billiar TR, et al. Decoding the multiple functions of ZBP1 in the mechanism of sepsis-induced acute lung injury. Commun Biol. 2024;7:1361. 39433574 10.1038/s42003-024-07072-x PMC11493966

[21] Fu Y, Gong T, Loughran PA, Li YH, Billiar TR, Liu YT, et al. Roles of TLR4 in macrophage immunity and macrophage-pulmonary vascular/lymphatic endothelial cell interactions in sepsis. Commun Biol. 2025;8:469. 40119011 10.1038/s42003-025-07921-3 PMC11928643

[22] Bain CC, MacDonald AS. The impact of the lung environment on macrophage development, activation and function: diversity in the face of adversity. Mucosal Immunol. 2022;15:223–34. 35017701 10.1038/s41385-021-00480-w PMC8749355

[23] Malainou C, Abdin SM, Lachmann N, Matt U, Herold S. Alveolar macrophages in tissue homeostasis, inflammation, and infection: evolving concepts of therapeutic targeting. J Clin Invest. 2023;133:e170501. 37781922 10.1172/JCI170501 PMC10541196

[24] Chen SZ, Saeed A, Liu Q, Jiang Q, Xu HZ, Xiao GG, et al. Macrophages in immunoregulation and therapeutics. Signal Transduct Target Ther. 2023;8:207. 37211559 10.1038/s41392-023-01452-1 PMC10200802

[25] Sutterwala FS, Ogura Y, Szczepanik M, Lara-Tejero M, Lichtenberger GS, Grant EP, et al. Critical role for NALP3/CIAS1/cryopyrin in innate and adaptive immunity through its regulation of caspase-1. Immunity. 2006;24:317–27. 16546100 10.1016/j.immuni.2006.02.004

[26] Wertman RS, Yost W, Herrmann BI, Bourne CM, Sorobetea D, Go CK, et al. Distinct sequential death complexes regulate pyroptosis and IL-1β release in response to Yersinia blockade of immune signaling. Sci Adv. 2024;10:eadl3629. 39058785 10.1126/sciadv.adl3629 PMC11277400

[27] Jaitin DA, Adlung L, Thaiss CA, Weiner A, Li BG, Descamps H, et al. Lipid-associated macrophages control metabolic homeostasis in a Trem2-dependent manner. Cell. 2019;178:686–98. 31257031 10.1016/j.cell.2019.05.054 PMC7068689

[28] Zhou CC, Guo Q, Lin JY, Wang M, Zeng Z, Li YJ, et al. Single-cell atlas of human ovaries reveals the role of the pyroptotic macrophage in ovarian aging. Adv Sci. 2024;11:eadl3629. 10.1002/advs.202305175 PMC10811476 38036420

[29] Lei YJ, VanPortfliet JJ, Chen YF, Bryant JD, Li Y, Fails D, et al. Cooperative sensing of mitochondrial DNA by ZBP1 and cGAS promotes cardiotoxicity. Cell. 2023;186:3013–32. 37352855 10.1016/j.cell.2023.05.039 PMC10330843

[30] Kuriakose T, Zheng M, Neale G, Kanneganti TD. IRF1 is a transcriptional regulator of ZBP1 promoting NLRP3 inflammasome activation and cell death during influenza virus infection. J Immunol. 2018;200:1489–95. 29321274 10.4049/jimmunol.1701538 PMC6483084

[31] Nassour J, Aguiar LG, Correia A, Schmidt TT, Mainz L, Przetocka S, et al. Telomere-to-mitochondria signalling by ZBP1 mediates replicative crisis. Nature. 2023;614:767–73. 36755096 10.1038/s41586-023-05710-8 PMC9946831

[32] Subramanian N, Natarajan K, Clatworthy MR, Wang Z, Germain RN. The adaptor MAVS promotes NLRP3 mitochondrial localization and inflammasome activation. Cell. 2013;153:348–61. 23582325 10.1016/j.cell.2013.02.054 PMC3632354

[33] Scicluna BP, van Vught LA, Zwinderman AH, Wiewel MA, Davenport EE, Burnham KL, et al. Classification of patients with sepsis according to blood genomic endotype: a prospective cohort study. Lancet Respir Med. 2017;5:816–26. 28864056 10.1016/S2213-2600(17)30294-1

[34] Zhang P, Gao C, Guo Q, Yang DX, Zhang GN, Lu H, et al. Single-cell RNA sequencing reveals the evolution of the immune landscape during perihematomal edema progression after intracerebral hemorrhage. J Neuroinflammation. 2024;21:140. 38807233 10.1186/s12974-024-03113-8 PMC11131315

[35] Kolter J, Döring CL, Sarout S, Baasch S, Steele L, Alsumati R, et al. Sensory neurons shape local macrophage identity via TGF-D signaling. Immunity. 2025;58:2556–73. 40914152 10.1016/j.immuni.2025.08.004

[36] Liu Y, Huo JL, Ren KD, Pan SK, Liu HD, Zheng YF, et al. Mitochondria-associated endoplasmic reticulum membrane (MAM): a dark horse for diabetic cardiomyopathy treatment. Cell Death Discov. 2024;10:148. 38509100 10.1038/s41420-024-01918-3 PMC10954771

[37] Missiroli S, Patergnani S, Caroccia N, Pedriali G, Perrone M, Previati M, et al. Mitochondria-associated membranes (MAMs) and inflammation. Cell Death Dis. 2018;9:329. 29491386 10.1038/s41419-017-0027-2 PMC5832426

[38] Sassano ML, Tyurina YY, Diometzidou A, Vervoort E, Tyurin VA, More S, et al. Endoplasmic reticulum-mitochondria contacts are prime hotspots of phospholipid peroxidation driving ferroptosis. Nat Cell Biol. 2025;27:902–17. 40514428 10.1038/s41556-025-01668-z PMC12173944

[39] Gong T, Zhang XD, Liu XL, Ye YF, Tian ZY, Yin S, et al. Exosomal Tenascin-C primes macrophage pyroptosis amplifying aberrant inflammation during sepsis-induced acute lung injury. Transl Res. 2024;270:66–80. 38604333 10.1016/j.trsl.2024.04.001

[40] Wu R, Zeng YN, Han JC, Li L, Xiong QM, Li SY, et al. Circulating FABP5 released by dying macrophages function as a DAMP to exacerbate septic inflammation. Cell Rep. 2025;44:116497. 41171759 10.1016/j.celrep.2025.116497

