# Supplementary material for: ZBP1-driven pyroptosis-associated alveolar macrophages exacerbate epithelial dysfunction in sepsis
Source: Cell Death Dis. 2026 Jun 26;17(1):793. doi: 10.1038/s41419-026-09043-y (PMC13562640; doi:10.1038/s41419-026-09043-y)
Supplement: Supplementary file 1 [file 41419_2026_9043_MOESM3_ESM.pdf]

Fig 2O

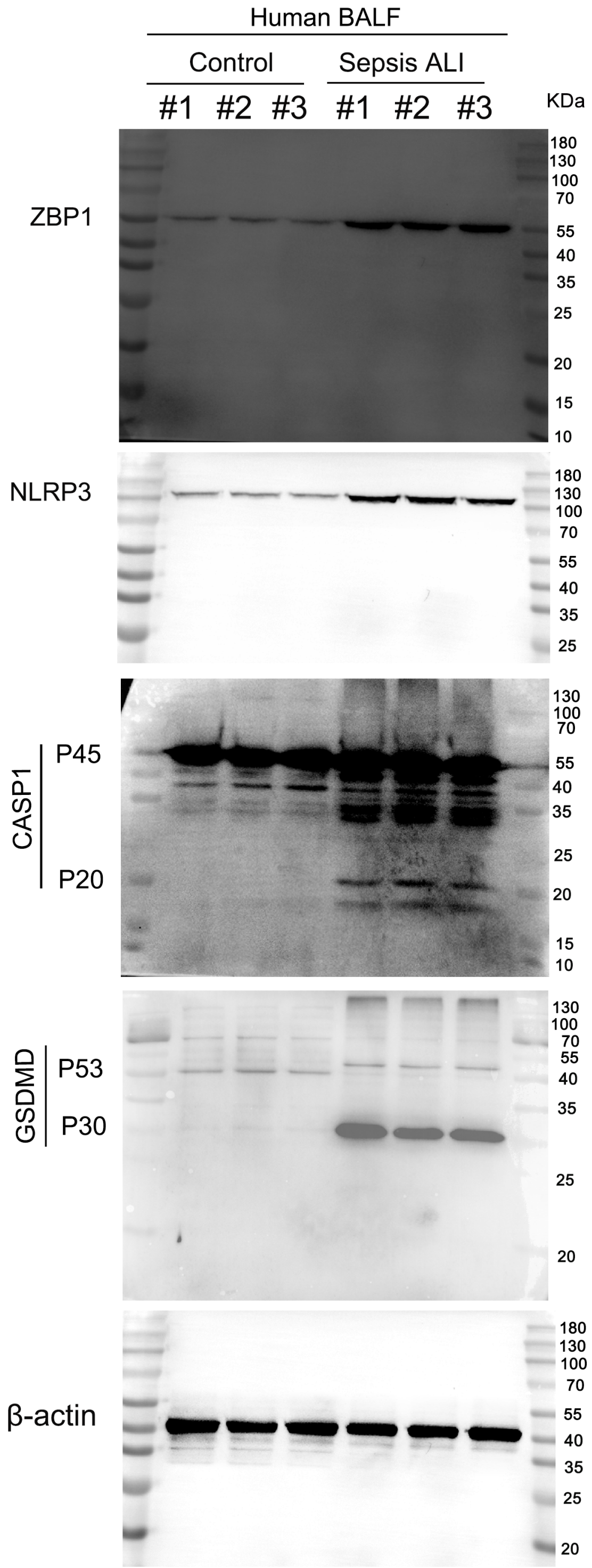
Human BALF
Control
Sepsis ALI
#1 #2 #3 #1 #2 #3
KDa
ZBP1
NLRP3
CASP1
P45
P20
GSDMD
P53
P30
β-actin
180
130
100
70
55
40
35
25
20
15
10

Fig 4K

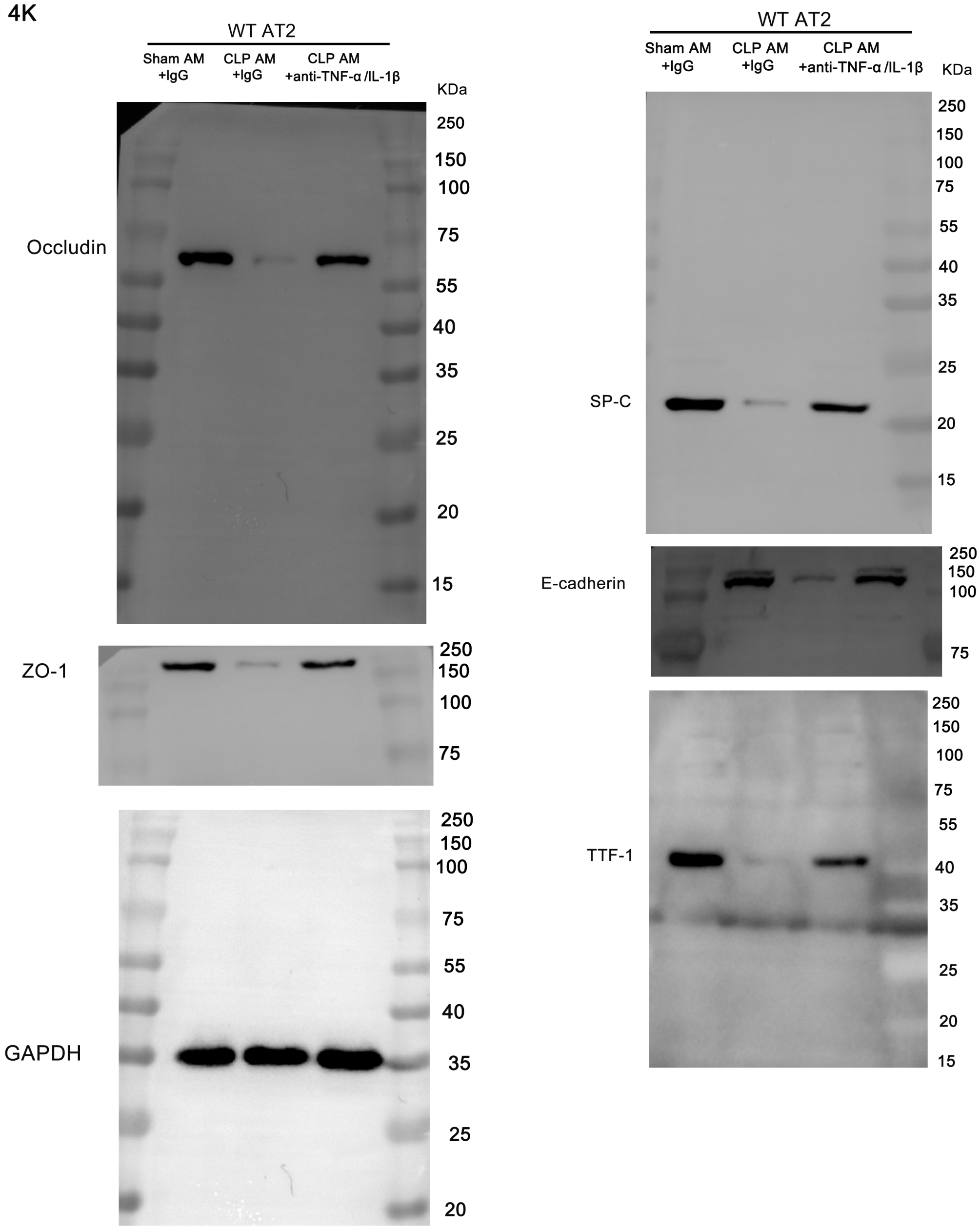

WT AT2
Sham AM +IgG
CLP AM +IgG
CLP AM +anti-TNF-α /IL-1β
KDa
Occludin
250
150
100
75
55
40
35
25
20
15
ZO-1
250
150
100
75
GAPDH
250
150
100
75
55
40
35
25
20
WT AT2
Sham AM +IgG
CLP AM +IgG
CLP AM +anti-TNF-α /IL-1β
KDa
SP-C
250
150
100
75
55
40
35
25
20
15
E-cadherin
250
150
100
75
TTF-1
250
150
100
75
55
40
35
25
20
15

Fig 6A

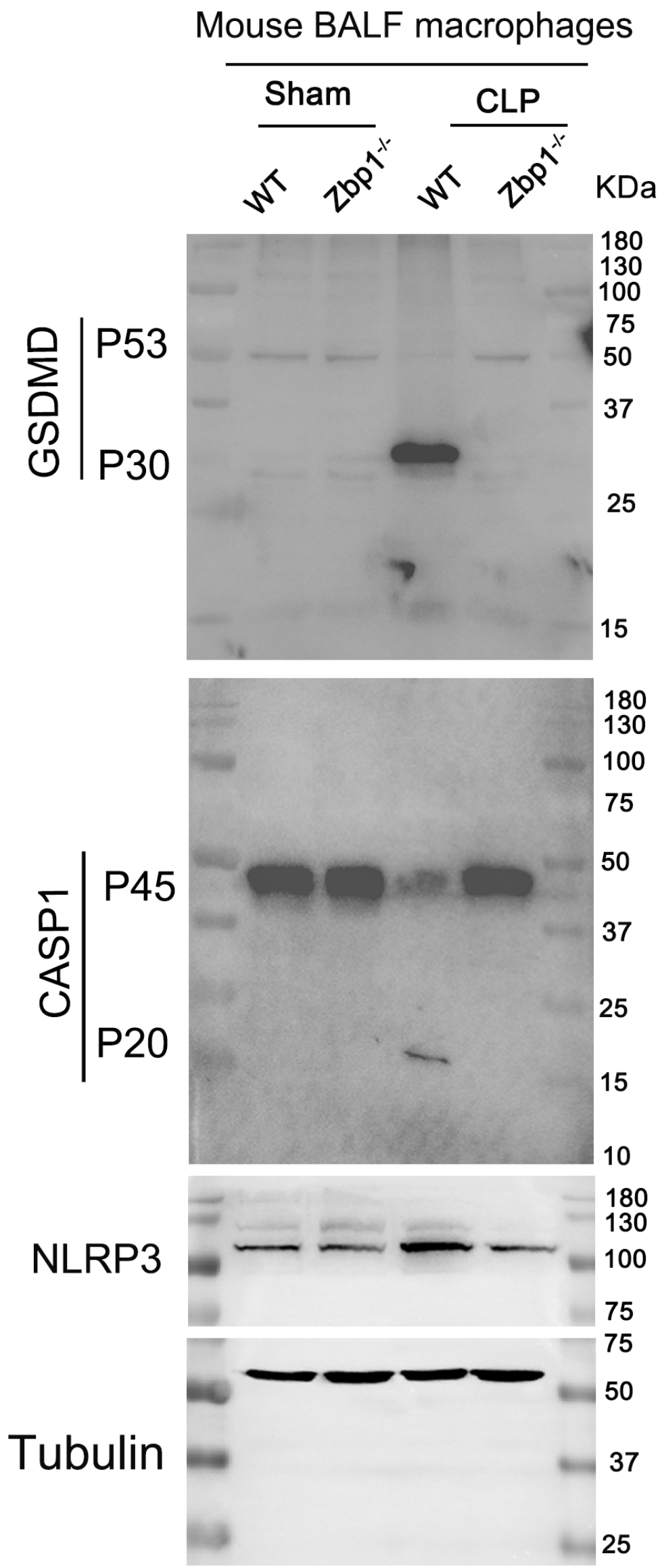
Mouse BALF macrophages
Sham
CLP
WT
Zbp1-/-
WT
Zbp1-/-
KDa
GSDMD
P53
P30
180
130
100
75
50
37
25
15
CASP1
P45
P20
180
130
100
75
50
37
25
15
10
NLRP3
180
130
100
75
Tubulin
75
50
37
25

Fig 6S

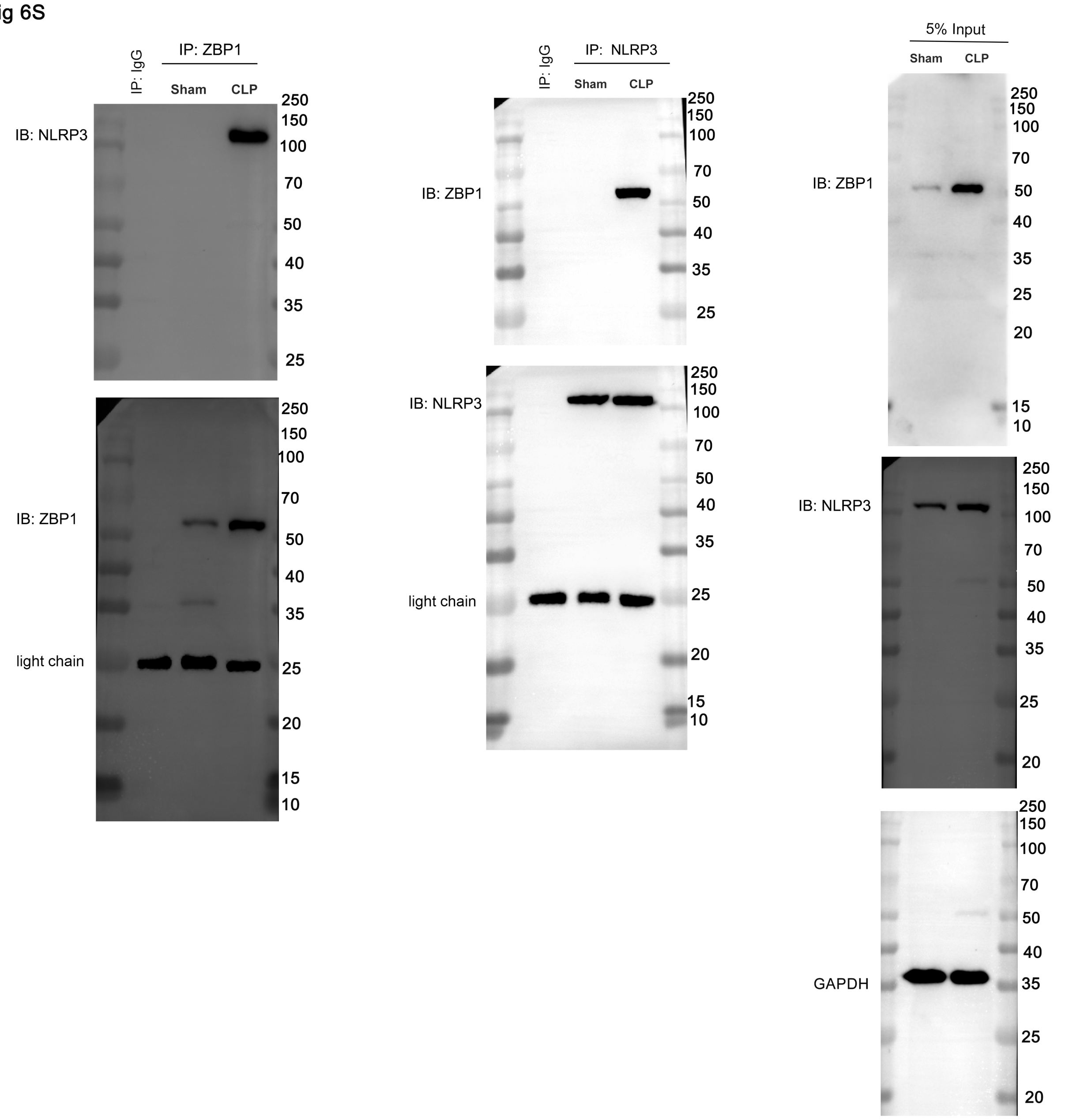
IP: IgG
IP: ZBP1
Sham
CLP
IB: NLRP3
IB: ZBP1
light chain
IP: IgG
IP: NLRP3
Sham
CLP
IB: ZBP1
IB: NLRP3
light chain
5% Input
Sham
CLP
IB: ZBP1
IB: NLRP3
GAPDH
250
150
100
70
50
40
35
25
20
15
10

Fig 7B

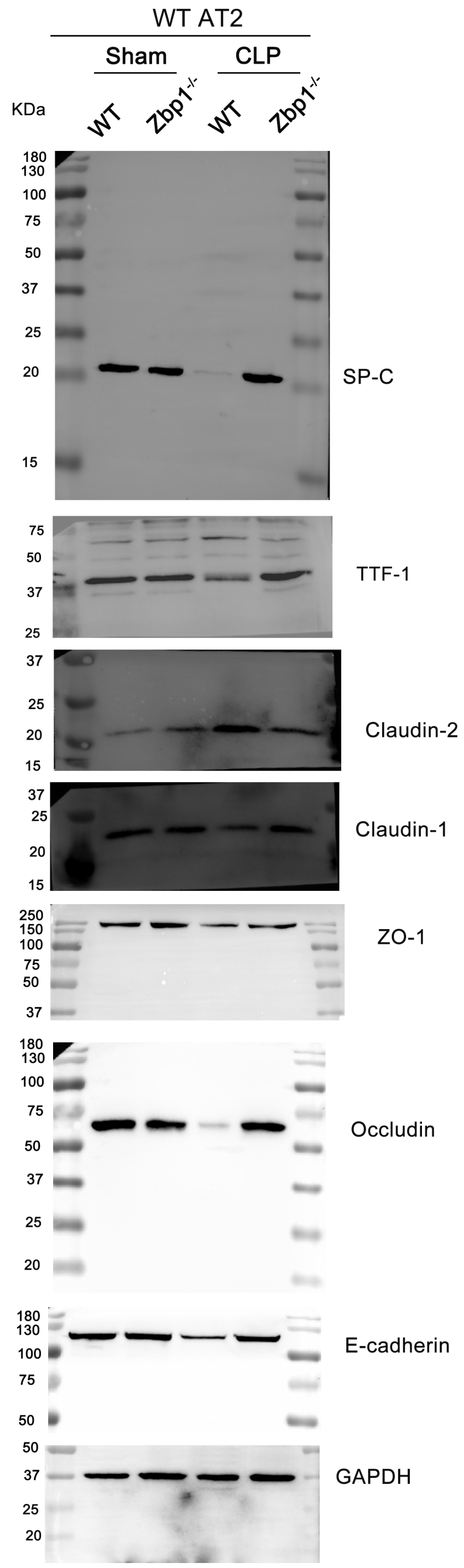

WT AT2
Sham
CLP
KDa
WT
Zbp1-/-
WT
Zbp1-/-
SP-C
TTF-1
Claudin-2
Claudin-1
ZO-1
Occludin
E-cadherin
GAPDH

Fig 7K

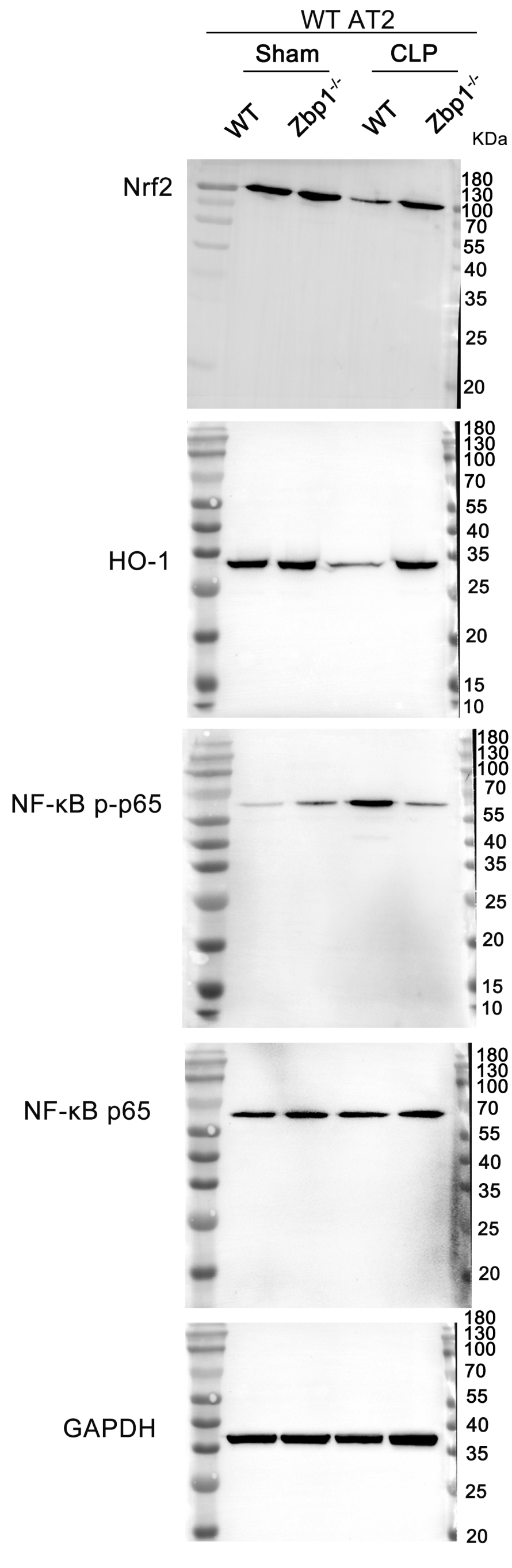

WT AT2
Sham
CLP
WT
Zbp1-/-
WT
Zbp1-/-
KDa
Nrf2
HO-1
NF-κB p-p65
NF-κB p65
GAPDH

Fig S2A

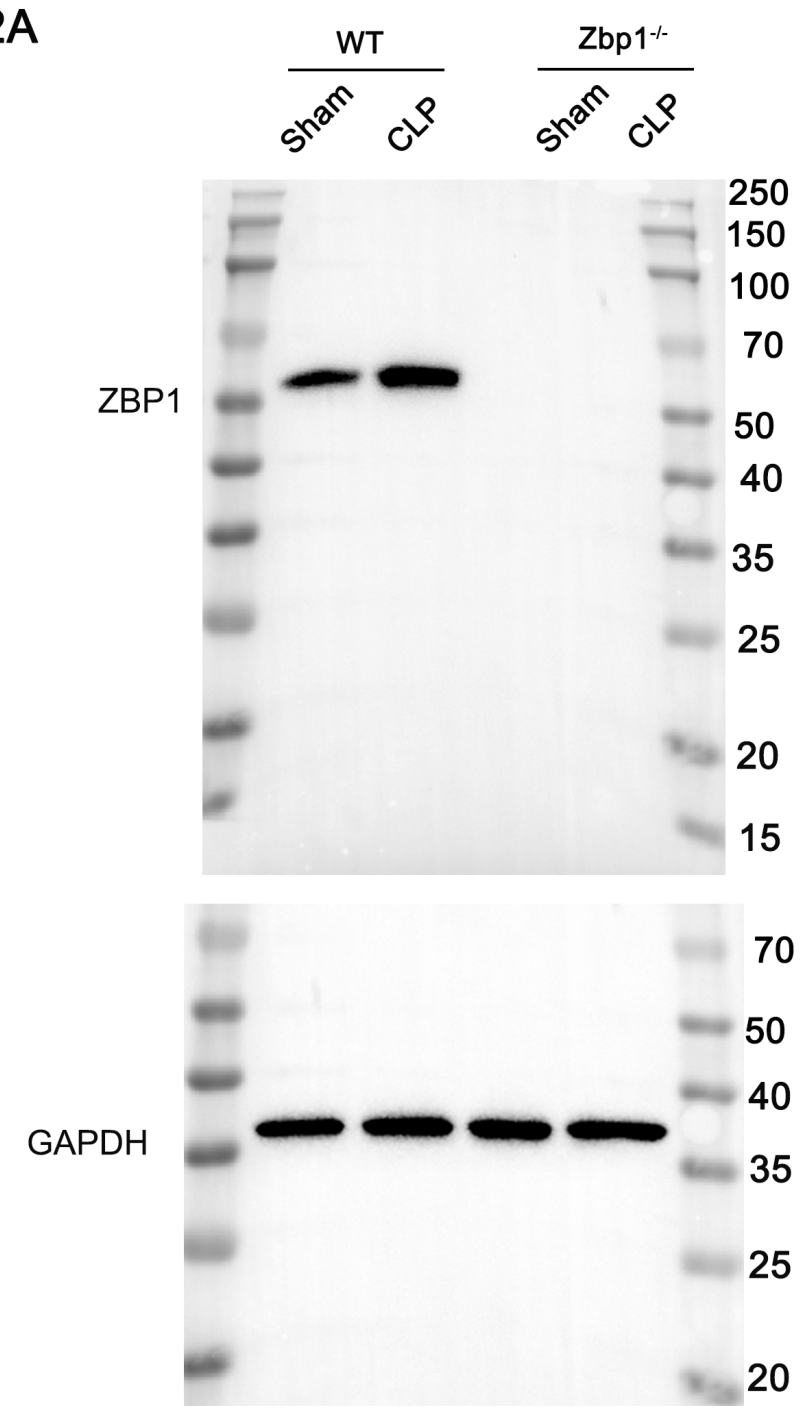
WT
Zbp1-/-
Sham
CLP
Sham
CLP
ZBP1
250
150
100
70
50
40
35
25
20
15
GAPDH
70
50
40
35
25
20
