# Supplementary material for: ZBP1-driven pyroptosis-associated alveolar macrophages exacerbate epithelial dysfunction in sepsis
Source: Cell Death Dis. 2026 Jun 26;17(1):793. doi: 10.1038/s41419-026-09043-y (PMC13562640; doi:10.1038/s41419-026-09043-y)
Supplement: Supplementary file 2 [file 41419_2026_9043_MOESM1_ESM.docx]

**Supplementary Figures**

**Figure S1. Pyroptotic NLRP3⁺ macrophages establish a dominant TNF/IL-1 signaling axis toward AT2 epithelial cells. B** Dot heatmap of key ligand-receptor pairs (rows) within TNF, IL-1, and CXCL families from macrophage subsets to AT2 cells (columns). Communication probability is color-coded; dot size denotes *P* < 0.01. **B-C** Violin plots of IL1B (B) and TNF (C) expression across macrophage subsets in SA and SD, showing maximal levels in NLRP3⁺ macrophages with significant elevation in SD (*****P* < 0.0001, ***P* < 0.01, ns, not significant).

**Figure S2. ZBP1 promotes macrophage pyroptosis and interacts with NLRP3 during sepsis-induced ALI.** A Western blot analysis of ZBP1 expression in BALF-derived alveolar macrophages from WT and *Zbp1*^-/-^ mice subjected to Sham or CLP. GAPDH was used as the loading control. B Quantification of pyroptotic bodies and mitochondria per cell based on transmission electron microscopy analysis of BALF-derived alveolar macrophages from WT and *Zbp1*^-/-^ mice after CLP. C Proximity ligation assay showing ZBP1-NLRP3 proximity signals in BALF-derived alveolar macrophages from Sham and CLP mice, with quantification of PLA signals per cell. D Immunofluorescence staining of ZBP1 and NLRP3 in BALF-derived alveolar macrophages from Sham and CLP mice, with representative enlarged images and line-scan colocalization analysis. Data are presented as mean ± SD. ***P* < 0.01, ****P* < 0.001. BALF, bronchoalveolar lavage fluid; AM, alveolar macrophage; CLP, cecal ligation and puncture; ALI, acute lung injury; PLA, proximity ligation assay.

**Figure S3. Identification and inflammatory characterization of epithelial cell subtypes in septic lungs.** A UMAP visualization showing epithelial cell subclusters identified in lung single-cell transcriptomic data, including AT2, AT1, club, and basal cells. B Heatmap showing the expression of representative marker genes used to annotate epithelial subtypes. C Heatmap showing the average expression of representative inflammatory chemokines and adhesion-related genes across epithelial subtypes.
