# Supplementary material for: ZBP1-driven pyroptosis-associated alveolar macrophages exacerbate epithelial dysfunction in sepsis
Source: Cell Death Dis. 2026 Jun 26;17(1):793. doi: 10.1038/s41419-026-09043-y (PMC13562640; doi:10.1038/s41419-026-09043-y)
Supplement: Supplementary file 3 [file 41419_2026_9043_MOESM2_ESM.docx]

**Supplementary Tables and Figures**

**Supplementary Tables**

**Table S1.** A list of antibodies used for Western blot, IHC staining, IF.

| Name of antibody | Cat. No | Company | Mol weight | Dilution  (WB/ IF) |
| --- | --- | --- | --- | --- |
| ZBP1 | sc-271483 | Santa Cruz | 55 kDa | 1:1000 (WB); 1:100 (IF) |
| NLRP3 | ab263899 | Abcam | 110 kDa | 1:1000 (WB); 1:100 (IF) |
| CASP1 | AG-20B-0042 | Adipogen | 45,20 kDa | 1:1000 (WB); 1:100 (IF) |
| GSDMD | ab209845 | Abcam | 53,34kDa | 1:1000 (WB) |
| SP-C | ab90716 | Abcam | 21 kDa | 1:1000 (WB) |
| TTF-1 | ab76013 | Abcam | 38 kDa | 1:1000 (WB) |
| Claudin-1 | ab180158 | Abcam | 22 kDa | 1:1000 (WB) |
| Claudin-2 | 32-5600 | Invitrogen | 22 kDa | 1:1000 (WB) |
| ZO-1 | 61-7300 | Invitrogen | 220 kDa | 1:1000 (WB) |
| Occludin | ab216327 | Abcam | 65 kDa | 1:1000 (WB) |
| E-cadherin | 14-3249-82 | Invitrogen | 120 kDa | 1:1000 (WB) |
| Nrf2 | #12721 | CST | 100 kDa | 1:1000 (WB) |
| HO-1 | ab68477 | Abcam | 32 kDa | 1:1000 (WB) |
| phospho-NF-κB p65 | #3033 | CST | 65 kDa | 1:1000 (WB) |
| NF-κB p65 | #8242 | CST | 65 kDa | 1:1000 (WB) |
| ASC | #67824 | CST | 22 kDa | 1:1000 (WB); 1:100 (IF) |
| GAPDH | 5174 | CST | 37 kDa | 1:1000 (WB) |
| Tubulin | sc-32293 | Santa Cruz | 55 kDa | 1:1000 (WB) |
| β-actin | #93473 | CST | 45 kDa | 1:1000 (WB) |

**Table S2.** A list of antibodies used for cytokine neutralization experiments

| Name of antibody | Clone | Company | Working concentration |
| --- | --- | --- | --- |
| Anti-mouse IL-1β antibody | B122 | eBioscience | 25 μg/mL |
| Anti-mouse TNF-α antibody | MP6-XT22 | BioLegend | 25 μg/mL |
| Armenian hamster IgG isotype control | eBio299Arm | eBioscience | 25 μg/mL |
| Rat IgG1, κ isotype control | RTK2071 | BioLegend | 25 μg/mL |
